# Association between atopic disorders and mental ill health: a UK-based retrospective cohort study

**DOI:** 10.1136/bmjopen-2024-089181

**Published:** 2025-05-31

**Authors:** Sonica Minhas, Joht Singh Chandan, Rebecca Knibb, Lavanya Diwakar, Nicola Adderley

**Affiliations:** 1 Institute of Applied Health Research, University of Birmingham, Birmingham, UK; 2 National Institute for Health and Care Research (NIHR) Birmingham Biomedical Research Centre, Birmingham, UK; 3 Aston University, Birmingham, UK; 4 University Hospitals of North Midlands NHS Trust, Stoke-on-Trent, UK

**Keywords:** MENTAL HEALTH, Allergy, EPIDEMIOLOGIC STUDIES, PUBLIC HEALTH, Primary Care

## Abstract

**Abstract:**

**Objective:**

To examine the mental ill health burden associated with allergic and atopic disorders, in a UK primary care cohort.

**Design:**

Population-based retrospective open cohort study.

**Setting:**

United Kingdom.

**Participants:**

2 491 086 individuals with primary-care recorded atopic disorder (food allergy, drug allergy, anaphylaxis, urticaria, allergic rhino-conjunctivitis) diagnosis were matched by sex, age (± 2 years), and socio-economic deprivation (Townsend quintile score) at index to 3 120 719 unexposed individuals. The mean age of exposed patients at cohort entry was 39.42 years (SD (SD) 23.65) compared with 35.81 years (SD 22.17) for unexposed patients.

**Main outcome measures:**

The primary outcome was a composite of mental ill health (severe mental illness, anxiety, depression, eating disorders, obsessive-compulsive disorder (OCD), and self-harm), identified using Read codes. Cox regression was used to estimate adjusted hazard ratios with 95% confidence intervals for the composite mental ill health outcome and each of the individual mental health disorders. Covariates adjusted for were age, sex, alcohol use, smoking status, body mass index (BMI), Townsend deprivation quintile score, asthma exposure, and eczema exposure at baseline.

**Results:**

Between first January 1995 to 31st January 2022, a total of 2 491 086 eligible individuals were identified with a primary care recorded diagnosis of atopic disease and were matched to 3 120 719 unexposed individuals. 229 124 exposed individuals developed a mental ill health outcome during the study period (incidence ratio (IR) 144.13 per 10 000 person-years) compared with 203 450 in the unexposed group (IR 117.82 per 10 000 person-years). This translated to an adjusted hazard ratio (aHR) of 1.16 (95% CI 1.15 to 1.17). Notably, the risk of anxiety was greatest, aHR 1.22 (95% CI 1.21 to 1.23). Our findings were robust to a sensitivity analysis, where individuals were also matched for asthma and eczema.

**Conclusion:**

There is an increased risk of mental ill health disorders among patients with diagnosis of an allergic and atopic disorders. There is a need to consider dual delivery of allergy and psychology services to optimise mental well-being among this cohort.

**STRENGTHS AND LIMITATIONS OF THIS STUDY**

- Large study sample of approximately 2.5 million patients which is representative of the UK general population with respect to demographics and comorbid conditions.
- Data (diagnoses for exposure and outcomes of interest) are derived from electronic primary care records reducing recall bias, as diagnoses are recorded at the time of consultation.
- Risk of heterogeneity in recording which can create misclassification bias, although this affects both the exposures and outcomes of interest.
- Due to poor recording in the primary care database, we are unable to perform stratification by disease severity status and account for confounders such as educational level and parental psychiatric history.

## Introduction

 Atopic and allergic disorders (food allergy, drug allergy, anaphylaxis, urticaria, and allergic rhino-conjunctivitis) are common chronic conditions globally and in the UK population,^1^ with significant associated morbidity.^2^ These disorders are caused by an aberrant immune system response, mediated by immunoglobulin E (IgE) antibiodies to otherwise innocuous antigens (allergens). The effects can range from mild and local responses (eg, mild allergic rhinitis) to life-threatening (eg, anaphylaxis).

Mental health disorders are also highly prevalent in the UK, with a 2014 survey showing that 1 in 6 people aged 16 years and older in England met the criteria for a common mental disorder (CMD).^3^ CMDs comprise of mental health disorders that interfere with daily functioning, but not insight or cognition. This includes different types of depression and anxiety, mixed depression-anxiety, phobias, obsessive compulsive disorder (OCD) and panic disorder.^4^ Mental health disorders are an important cause of disability and are a risk factor for premature mortality.^5 6^ Severe mental illness (SMI), by contrast, is a subset of mental health disorders characterised by a greater degree of functional and occupational impairment, limiting major life activities and typically includes psychoses and bipolar disorder.^7^ Data from England in 2018 to 2020 shows that people with SMI are five times more likely to die prematurely (before age 75 years) than those without an SMI.^8^

Increasingly, research suggests a relationship between atopic disease and mental health conditions.^9^ Epidemiological research has shown associations between allergic rhinitis and mood disorders,^10 11^ suicidal ideation,^12^ and anxiety disorders.^13^ Although less well studied, some research has shown that urticaria is also associated with reduced quality of life and greater symptoms of depression and anxiety.^14 15^ Food allergy has been associated with reduced quality of life, anxiety and depression,^16 17^ as has anaphylaxis.^18^ The majority of this research has been in non-UK cohorts and is limited by small sample sizes and cross-sectional design. Given the high prevalence of both atopic and mental health disorders, understanding any association between the two is vital for improving patient care and for targeting interventions to reduce the burden associated with both. Using UK primary care data, we investigated the associations between atopic disorders and the subsequent risk of developing mental ill health disorders. As there is already a strong evidence base for asthma, allergic rhinitis and atopic dermatitis,^9^,^11^ we focused our analysis on food allergy, drug allergy, anaphylaxis, urticaria, allergic rhino-conjunctivitis.

## Methods

### Study design, data source, and population

A UK population-based, retrospective, open cohort study was undertaken using data from the IQVIA Medical Research Database (IMRD) from first January 1995 to 28th February 2021. The IMRD UK database contains de-identified electronic medical records from UK primary care general practices using the Vision software system. It is nationally representative of the UK population, with respect to demographic structure and the prevalence of common comorbidities.^19 20^ Patient data regarding symptoms, examination findings, and diagnoses is recorded using Read codes^21^ - a hierarchical clinical coding system used in UK primary care.

The ‘Data extraction for epidemiological research (DExtER)’ tool^22^ was used to facilitate data extraction, transformation and loading in this study. The data analysis and reporting adhered to the Strengthening the Reporting of Observational Studies in Epidemiology (STROBE) statement (online supplemental appendix A).^23^

To ensure accurate coding and reduce underreporting of outcomes, general practices were only eligible for inclusion from the later of the following two dates: 1 year after the installation of the Vision software system or the date acceptable mortality recording (AMR) was achieved.^24^ AMR date is determined by the comparison between recording of death by practices and the predicted mortality, using national data, accounting for the demographic of a practices’ catchment area. The AMR date is defined as the point where the national and practice mortality rates align.

### Exposure and outcome definition

The aim of this study was to compare the risk of mental ill health (composite measure: defined through Read codes describing depression, anxiety, severe mental illness [psychosis, bipolar disorder or schizophrenia], eating disorders, OCD, and self-harm) in exposed patients (Read codes for food allergy, drug allergy, anaphylaxis, urticaria, allergic rhino-conjunctivitis) with unexposed patients (those without such codes). For this analysis, self-harm includes any deliberate act of inflicting harm on oneself, regardless of suicidal intent. Codes for allergic and atopic disorders and mental ill health were selected with the help of general health practitioners, public health clinicians and immunologists to define the exposed cohort (see Appendix B for the codes used in this study). The primary outcome of interest was a composite of mental ill health but each of the aforementioned mental health disorders was also analysed separately. Patients with mental health disorders at baseline were excluded from the study.

Individuals with atopic disorders were matched to up to four unexposed individuals randomly selected from the remaining pool of eligible patients, by general practice, age (± 2 years), sex and Townsend deprivation quintile score^25^ at baseline. The Townsend deprivation quintile score is a measure of socioeconomic status derived from national census data, including variables such as home ownership and employment.^26^ A higher score correlates to greater socio-economic deprivation.

### Follow-up period

Index date for patients in the exposed group was defined as the date of first Read code relating to atopic disorder diagnosis (incident cases) or the date of eligibility of cohort entry for those with pre-existing recoded Read code (prevalent cases). To prevent immortal time bias,^24^ matched unexposed patients were assigned the same index date. An open cohort design allows patients to be enrolled at different timepoints, and for each patient to contribute person-years of follow-up from the point of entry (index date) to the point of exit (exit date). Exit date was defined as the earliest of (1) the outcome, (2) death, (3) patient left the general practice, (4) last date of data collection from the practice, or (5) study end date.

### Study covariates

Co-variates adjusted for in the analysis were: age, sex, alcohol use, smoking status, body mass index (BMI), Townsend deprivation quintile score, asthma exposure, and eczema exposure at baseline. These co-variates were selected due to their potential independent relationship with the development of mental ill health.^27 28^ Our matching criteria included age, sex, and Townsend deprivation quintile score, however we also included these as covariates in the study models to account for any residual confounding.

BMI was calculated as weight in kilograms divided by height in metres squared and categorised as per the WHO criteria (underweight<18·5 kg/m2, normal 18·5–24·9kg/m^2^, overweight 25·0–29·9kg/m^2^, obese>30·0kg/m^2^). Smoking status (current smoker, non-current smoker, not available) and alcohol use (current drinker, non-current drinker, not available) were self-reported.

### Statistical analysis

STATA version 18^29^ was used for all analyses. Missing data was included as a separate missing category and was included in the regression analysis, in keeping with previously published work.^30^,^32^

Crude incidence rates (IR) were calculated for the exposed and unexposed groups by dividing the number of outcomes by person-years. A Cox proportional hazards regression model was used to derive unadjusted and adjusted hazard ratios (HR) with 95% CIs describing the association between atopic disorders and the risk of mental ill health (composite outcome) and then for each mental health disorder included in the study individually. Statistical significance was set at p<0.05. A sensitivity analysis was undertaken by matching participants for asthma and eczema exposure.

### Patient and public involvement

Patients and/or the public were not involved in setting the research or interpretation and write up of results. This was deemed unnecessary as all patient related data used was anonymised.

## Results

In this study, 2 491 086 individuals were identified with a primary care recorded diagnosis of atopic disease and they were matched to 3 120 719 unexposed individuals. All data describing baseline characteristics are outlined in table 1. The exposed cohort had a median follow-up period of 5.10 person years (IQR 2.02–9.68) vs 4.11 (1.59–8.37) in the unexposed cohort. Due to matching, the mean age at cohort entry and sex distribution were similar, although exposed individuals were more likely to be female than unexposed individuals (54.64 vs 49.98%). Data on BMI, smoking status, drinking status, Townsend index, and ethnicity were missing in both groups. Where recorded, data on smoking status, drinking status, and Townsend index were similar between the groups. However, individuals in the exposed group were more likely to be obese (13.99% vs 9.94%) and of white ethnicity (42.19% vs 35.72%) compared with those in the unexposed group.

**Table 1:** Baseline characteristics

	Exposed	Unexposed
Number of patients (n)	2 491 086	3 120 719
Number of patients with asthma diagnosis (n)	472 984	289 969
Number of patients with eczema diagnosis (n)	455 348	367 786
Median (IQR) follow-up period (person years)	5.10 (2.02–9.68)	4.11 (1.59–8.37)
Mean (SD) age at cohort entry (years)	39.42 (23.65)	35.81 (22.17)
Sex (n)		
Male	1 129 954	1 561 074
Female	1 361 132	1 559 645
Body mass index, n (%)		
Underweight (<18.5 kg/m^2^)	40 490 (1.63)	52 235 (1.67)
Normal (18.5–24.9 kg/m^2^)	687 723 (27.61)	823 574 (26.39)
Overweight (25.0–29.9 kg/m^2^)	541 784 (21.75)	559 329 (17.92)
Obese (>30.0 kg/m^2^)	348 395 (13.99)	310 123 (9.94)
Not available	872 694 (35.03)	1 375 458 (44.08)
Smoking status, n (%)		
Current smoker	348 532 (13.99)	474 771 (15.21)
Non-current smoker	1 574 697 (63.21)	1 681 583 (53.88)
Not available	567 857 (22.80)	964 365 (30.90)
Drinking status, n (%)		
Current Drinker	1 266 898 (50.86)	1 394 975 (44.70)
Non-current drinker	370 169 (14.86)	382 726 (12.26)
Not available	854 019 (34.28)	1 343 018 (43.04)
Townsend index, n (%)		
(Least deprived) 1	535 228 (21.49)	657 580 (21.07)
2	464 489 (18.65)	552 877 (17.72)
3	453 156 (18.19)	559 350 (17.92)
4	388 862 (15.61)	487 367 (15.62)
5	262 946 (10.56)	327 038 (10.48)
Not available	386 405 (15.51)	536 507 (17.19)
Ethnicity, n (%)		
White	1 050 939 (42.19)	1 114 783 (35.72)
Black	45 742 (1.84)	51 975 (1.67)
South Asian	66 060 (2.65)	74 028 (2.37)
Mixed	16 818 (0.68)	19 858 (0.64)
Other	37 641 (1.51)	54 616 (1.75)
Missing	1 273 886 (51.14)	1 805 459 (57.85)

During the study period, there were 229 124 recorded outcomes of mental ill health in the exposed group (IR 144.13 per 10 000 person-years) compared with 203 450 in the unexposed group (IR 117.82 per 10 000 person-years); (table 2). This translated to an aHR of 1.16 (95% CI 1.15 to 1.17). The risk of anxiety (aHR 1.22, 95% CI 1.21 to 1.23), depression (aHR 1.15, 95% CI 1.14 to 1.16), eating disorders (aHR 1.06, 95% CI 1.01 to 1.11), OCD (aHR 1.20, 95% CI 1.14 to 1.26), and self-harm (aHR 1.02, 95% CI 1.00 to 1.04) were greater in the exposed group compared with the unexposed group. By contrast, there was no difference in the risk of SMI between the two groups, aHR 0.99 (9% CI 0.95 to 1.03).

**Table 2:** Hazard ratios with 95% confidence intervals (CI) for mental ill health among patients with any atopic or allergic disorder compared with matched unexposed individuals

		Number of outcomes	Person years	Incidence rate (per 10 000 person years)	Unadjusted HR	Adjusted HR*
Composite	Exposed	229 124	15 900 000	144.13	1.23 (95% CI 1.23 to 1.24, p<0.001)	1.16 (95% CI 1.15 to 1.17, p<0.001)
	Unexposed	203 450	17 300 000	117.82		
SMI	Exposed	5581	17 200 000	32.46	1.00 (95% CI 0.97 to 1.04, p 0.895)	0.99 (95% CI 0.95 to 1.03, p 0.646)
	Unexposed	5979	18 400 000	32.51		
Anxiety	Exposed	93 848	16 700 000	56.210	1.30 (95% CI 1.29 to 1.31, p<0.001)	1.22 (95% CI 1.21 to 1.23, p<0.001)
	Unexposed	77 479	18 000 000	43.05		
Depression	Exposed	138 658	16 400 000	84.59	1.25 (95% CI 1.24 to 1.26, p<0.001)	1.15 (95% CI 1.14 to 1.16, p<0.001)
	Unexposed	121 244	17 700 000	68.48		
Eating disorders	Exposed	4188	17 200 000	2.44	1.06 (95% CI 1.01 to 1.10, p 0.013)	1.06 (95% CI 1.01 to 1.11, p 0.01)
	Unexposed	4310	18 400 000	2.34		
OCD	Exposed	3565	17 200 000	2.07	1.20 (95% CI 1.15 to 1.26, p<0.001)	1.20 (95% CI 1.14 to 1.26, p<0.001)
	Unexposed	3163	18 400 000	1.72		
Self harm	Exposed	21 959	17 100 000	12.83	0.99 (95% CI 0.98 to 1.01, p 0.518)	1.02 (95% CI 1.00 to 1.04, p 0.032)
	Unexposed	23 619	18 300 000	12.90		

* Adjusted HR: adjusted for age, sex, alcohol use, smoking status, body mass index (BMI), Townsend deprivation quintile score, asthma, and eczema at baseline.

OCD, obsessive-compulsive disorder; SMI, severe mental illness.

Table 3A–E describe the risk of mental ill health outcomes associated with each type of atopic disorder included in the exposure for this study (food allergy, drug allergy, anaphylaxis, urticaria, and allergic rhinitis respectively). All individual atopic disorders were associated with greater risk of mental ill health compared with non-exposed individuals: food allergy (aHR 1.07, 95% CI 1.03 to 1.11); drug allergy (aHR 1.28, 95% CI 1.27 to 1.29); anaphylaxis (aHR 1.43, 95% CI 1.32 to 1.54); urticaria (aHR 1.15, 95% CI 1.14 to 1.17); allergic rhinitis (aHR 1.12, 95% CI 1.12 to 1.14) (table 3A–E).

**Table 3:** Hazard ratios with 95% confidence intervals (CI) for mental ill health among patients with specific atopic and allergic disorders

		Number of outcomes	Person years	Incidence rate (per 10 000 person years)	Unadjusted HR	Adjusted HR
(A) Food allergy						
Composite	Exposed	6955	583 190	119.26	1.21 (95% CI 1.17 to 1.25, p<0.001)	1.07 (95% CI 1.03 to 1.11, p<0.001)
	Unexposed	6335	648 193	97.73		
SMI	Exposed	180	617 205	2.92	1.18 (95% CI 0.95 to 1.46, p 0.129)	1.13 (95% CI 0.90 to 1.42, p 0.286)
	Unexposed	165	677 620	2.44		
Anxiety	Exposed	2908	604 387	48.12	1.33 (95% CI 1.26 to 1.40, p<0.001)	1.15 (95% CI 1.09 to 1.22, p<0.001)
	Unexposed	2355	667 832	35.26		
Depression	Exposed	3692	597 935	61.75	1.18 (95% CI 1.13 to 1.24, p<0.001)	1.03 (95% CI 0.98 to 1.09, p 0.184)
	Unexposed	3425	660 867	51.83		
Eating disorders	Exposed	263	616 739	4.26	1.20 (95% CI 1.01 to 1.43, p 0.042)	1.09 (95% CI 0.91 to 1.32, p 0.351)
	Unexposed	240	677 337	3.54		
OCD	Exposed	188	617 035	3.05	1.27 (95% CI 1.03 to 1.57, p 0.028)	1.12 (95% CI 0.89 to 1.41, p 0.341)
	Unexposed	158	677 684	2.33		
Self harm	Exposed	917	614 055	14.93	1.01 (95% CI 0.92 to 1.10, p 0.891)	0.95 (95% CI 0.86 to 1.05, p 0.335)
	Unexposed	970	674 407	14.38		
(B) Drug allergy						
Composite	Exposed	136 246	8 629 804	157.88	1.35 (95% CI 1.33 to 1.36, p<0.001)	1.28 (95% CI 1.27 to 1.29, p<0.001)
	Unexposed	104 501	8 773 298	119.11		
SMI	Exposed	3467	9 429 027	3.68	1.07 (95% CI 1.02 to 1.13, p 0.004)	1.07 (95% CI 1.02 to 1.12, p 0.007)
	Unexposed	3245	9 364 852	3.47		
Anxiety	Exposed	55 508	9 120 391	60.86	1.43 (95% CI 1.41 to 1.45, p<0.001)	1.36 (95% CI 1.34 to 1.37, p<0.001)
	Unexposed	39 114	9 159 493	42.70		
Depression	Exposed	85 637	8 920 011	96.01	1.35 (95% CI 1.34 to 1.37, p<0.001)	1.27 (95% CI 1.25 to 1.28, p<0.001)
	Unexposed	64 926	8 990 436	72.22		
Eating disorders	Exposed	1834	9 434 611	1.94	1.09 (95% CI 1.02 to 1.16, p 0.011)	1.12 (95% CI 1.05 to 1.20, p 0.001)
	Unexposed	1711	9 371 394	1.83		
OCD	Exposed	1663	9 434 811	1.76	1.34 (95% CI 1.24 to 1.44, p<0.001)	1.37 (95% CI 1.27 to 1.47, p<0.001)
	Unexposed	1241	9 372 528	1.32		
Self harm	Exposed	10 780	9 390 771	11.48	1.13 (95% CI 1.10 to 1.16, p<0.001)	1.18 (95% CI 1.15 to 1.22, p<0.001)
	Unexposed	9531	9 333 037	10.21		
(C) Anaphylaxis						
Composite	Exposed	1773	100 899	175.72	1.57 (95% CI 1.46 to 1.69, p<0.001)	1.43 (95% CI 1.32 to 1.54, p<0.001)
	Unexposed	1229	108 556	113.21		
SMI	Exposed	33	111 340	2.96	0.99 (95% CI 0.61 to 1.58, p 0.951)	0.93 (95% CI 0.57 to 1.54, p 0.792)
	Unexposed	36	115 380	3.12		
Anxiety	Exposed	769	107 135	71.78	1.76 (95% CI 1.57 to 1.97, p<0.001)	1.59 (95% CI 1.41 to 1.79, p<0.001)
	Unexposed	460	112 994	40.71		
Depression	Exposed	1050	104 749	100.24	1.51 (95% CI 1.38 to 1.66, p<0.001)	1.36 (95% CI 1.23 to 1.50, p<0.001)
	Unexposed	748	111 314	67.20		
Eating disorders	Exposed	31	111 337	2.78	1.08 (95% CI 0.65 to 1.78, p 0.777)	0.89 (95% CI 0.51 to 1.56, p 0.693)
	Unexposed	30	115 429	2.60		
OCD	Exposed	32	111 288	2.88	2.04 (95% CI 1.13 to 3.68, p 0.018)	2.37 (95% CI 1.28 to 4.40, p 0.006)
	Unexposed	17	115 524	1.47		
Self harm	Exposed	196	110 613	17.72	1.94 (95% CI 1.53 to 2.47, p<0.001)	1.83 (95% CI 1.42 to 2.36, p<0.001)
	Unexposed	105	115 123	9.12		
(D) Urticaria						
Composite	Exposed	43 001	2.966,951	144.93	1.25 (95% CI 1.23 to 1.27, p<0.001)	1.15 (95% CI 1.14 to 1.17, p<0.001)
	Unexposed	35 624	3 060 309	116.41		
SMI	Exposed	863	3 231 215	2.67	0.91 (95% CI 0.83 to 0.99, p 0.035)	0.87 (95% CI 0.79 to 0.96, p 0.004)
	Unexposed	950	3 261 340	2.91		
Anxiety	Exposed	18 675	3 123 901	59.78	1.33 (95% CI 1.30 to 1.36, p<0.001)	1.23 (95% CI 1.20 to 1.25, p<0.001)
	Unexposed	14 118	3 188 116	44.28		
Depression	Exposed	24 988	3 072 521	96.01	1.27 (95% CI 1.25 to 1.29, p<0.001)	1.15 (95% CI 1.13 to 1.17, p<0.001)
	Unexposed	20 251	3 143 972	72.22		
Eating disorders	Exposed	1090	3 229 622	3.38	1.12 (95% CI 1.03 to 1.22. p 0.008)	1.13 (95% CI 1.03 to 1.23, p 0.007)
	Unexposed	992	3 261 134	3.04		
OCD	Exposed	786	3 230 785	2.43	1.24 (95% CI 1.12 to 1.38, p<0.001)	1.20 (95% CI 1.08 to 1.34, p<0.001)
	Unexposed	630	3 262 586	1.93		
Self harm	Exposed	5105	3 209 184	15.91	1.03 (95% CI 0.99 to 1.07, p 0.162)	1.03 (95% CI 0.99 to 1.07, p 0.176)
	Unexposed	4942	3 241 833	15.24		
(E) Allergic rhinitis						
Composite	Exposed	12 576	725 508	173.34	1.21 (95% CI 1.20 to 1.22, p<0.001)	1.12 (95% CI 1.12 to 1.14, p<0.001)
	Unexposed	8368	650 841	128.57		
SMI	Exposed	239	812 716	2.94	0.95 (95% CI 0.90 to 1.01, p 0.101)	0.96 (95% CI 0.90 to 1.02, p 0.149)
	Unexposed	208	701 764	2.96		
Anxiety	Exposed	5588	775 154	75.96	1.30 (95% CI 1.29 to 1.32, p<0.001)	1.21 (95% CI 1.19 to 1.23, p<0.001)
	Unexposed	3400	682 732	49.80		
Depression	Exposed	7308	760 744	96.06	1.22 (95% CI 1.21 to 1.24, p<0.001)	1.12 (95% CI 1.11 to 1.14, p<0.001)
	Unexposed	4824	672 055	71.78		
Eating disorders	Exposed	349	811 814	4.30	1.09 (95% CI 1.03 to 1.16, p 0.004)	1.06 (95% CI 0.99 to 1.13, p 0.061)
	Unexposed	234	701 573	3.34		
OCD	Exposed	263	812 322	3.24	1.26 (95% CI 1.18 to 1.35, p<0.001)	1.23 (95% CI 1.14 to 1.31, p<0.001)
	Unexposed	142	702 130	2.02		
Self harm	Exposed	1430	805 392	17.76	0.93 (95% CI 0.90 to 0.95, p<0.001)	0.93 (95% CI 0.91 to 0.96, p<0.001)
	Unexposed	1212	696 241	17.41		

* Adjusted HR: adjusted for age, sex, alcohol use, smoking status, body mass index (BMI), Townsend deprivation quintile score, asthma, and eczema at baseline.

OCD, obsessive-compulsive disorder; SMI, severe mental illness.

Notably, our results demonstrated that food allergy and drug allergy exposure were associated with increased risk of all mental health outcomes. Individuals with anaphylaxis exposure had the greatest risk of anxiety (aHR 1.59, 95% CI 1.41 to 1.79), OCD (aHR 2.37, 95% CI 1.28 to 4.40), and depression (aHR 1.36, 95% CI 1.23 to 1.50); (table 3c).

We carried out a sensitivity analysis, matching both groups for asthma and eczema in addition to other baseline characteristics (online supplemental table S1). The findings of the sensitivity analysis were similar to that of the main cohort. There was an increased risk of all mental ill health outcomes in those with atopic disease exposure compared with those without, aHR 1.16 (95% CI 1.15 to 1.16). The risk of all individual mental health outcomes except SMI was increased, reflecting the main cohort analysis: SMI (aHR 0.99, 95% CI 0.96 to 1.03); anxiety (aHR 1.21, 95% CI 1.20 to 1.23); depression (aHR 1.15, 95% CI 1.14 to 1.16); eating disorders (aHR 1.06, 95% CI 1.01 to 1.10); OCD (aHR 1.18, 95% CI 1.13 to 1.24); self-harm (aHR 1.03; 95% CI 1.01 to 1.05).

Online supplemental tables S3–S7 describe the risk of mental ill health outcomes associated with each type of atopic disorder, in the sensitivity analysis, with patients also matched for asthma and eczema.

## Discussion

### Principal findings

In this population-based UK retrospective cohort study, we found that individuals with primary care recorded exposure to atopic disease had a 16% higher risk of having a subsequent mental health diagnosis, compared with those with no such exposure. Analysis of each individual mental health outcome showed a positive association with atopy exposure, except SMI. These findings were robust to a sensitivity analysis, suggesting that the impact was related to atopy rather than eczema or asthma.

Consideration of individual atopic and allergic disorders showed that individuals with anaphylaxis exposure had the greatest risk of subsequent mental health diagnosis, with 43% increased risk compared with those without. They were most likely to be diagnosed with OCD, anxiety, or depression (137%, 59% and 36% increased risk respectively). Notably, only drug allergy and food allergy exposure conferred an increased risk of SMI.

### Comparison with previous studies

The evidence base for atopic disorders (other than asthma, atopic dermatitis, and allergic rhinitis) and their relationship with mental health disorders is still emerging, especially for diagnoses other than depression and anxiety. Our study expands on the existing global literature. It supports the findings of previous studies demonstrating a relationship between atopic disorders and common mental health disorders (depression and anxiety). Although the strength of associations previously found varies across the conditions being assessed and the majority of previous studies explored mental health outcomes in those with allergic rhinitis, asthma, and/or eczema. Some evidence has also linked allergy, eating disorders,^33^ attention deficit hyperactivity disorder (ADHD) and suicides.^34^ Our study has examined these associations in a large sample, representative of the general UK population where previous studies have often included small sample sizes of clinical population, which may differ in their demographics from the general population.

Previous research has indicated a strong association between food allergy and anxiety, which could be driven by the fear of a life-threatening reaction.^17^ Our study’s findings provide support to this association. Individuals with food allergy in our study demonstrated a 15% increase in anxiety (aHR 1.15, 95% CI 1.09 to 1.22); (table 3a).

Previous research indicates the relationship between atopic disorders and mental health disorders appears to be bidirectional. Some studies have shown that individuals with depression are more likely to have a concomitant diagnosis of allergies, including non-food allergies.^35^,^38^ A twin study from Finland suggested the possibility of a genetic link between allergy and depression.^39^ Birth cohort studies from northern Finland have demonstrated strong association between depression and allergy, especially in females.^40 41^ One study demonstrated that females with atopy had an 80% increased risk of developing depression (aOR 1.8, 95% CI 1.2 to 2.6).^41^

### Potential underlying mechanisms

It has been suggested that patients with allergies and mental health disorders have similar disruptions in immune pathways.^42^ Depression and anxiety can enhance the inflammatory Th2 and Th17 pathways^34^ which are also implicated in the aetiology of allergy.^43^ Indeed, there is clinical evidence to suggest that this relationship could be bi-directional - that is, having a diagnosis of either allergy or a mental health disorder can increase the risk of the other (Zhang et al., 2022). In further support of this hypothesis, medication for depression has been shown to improve allergy symptoms^42^ and vice-versa.^44^ Poor allergy management can worsen outcomes too: a Canadian study found increased tics, anxiety and disruptive behaviour patterns in children aged 5–10 years receiving hydroxyzine, a first-generation antihistamine.^45^

The data on the relationship between severity of allergy and mental health disease are less clear. One study suggested that the likelihood of mental health disease increases with increased severity of atopic dermatitis,^46^ but there are no data on the risks related to other allergic conditions. One hypothesis is that immunological and inflammatory responses associated with atopy may predispose to increased risk of mental health disorders, particularly depression.^47^ In sensitised individuals, exposure to an allergen results in IgE-mediated activation of mast cells, causing mass secretion of chemicals such as histamine and cytokines (eg, interleukin-4 (IL-4) and IL-13). Recent evidence suggests that these cytokines can alter neurotransmitter systems, contributing to the development of psychiatric disorders.^48^ Pro-inflammatory cytokine exposure has been shown to induce symptoms of anhedonia, lethargy, sleep disturbance, and anorexia- all of which are established symptoms of depression.^49 50^ Many pathophysiologic pathways in the nervous system are posited to be involved in depression, including the monoaminergic pathway, dysfunction of the hypothalamic-pituitary axis (HPA), and glutamate transmission.^48 51^

A second hypothesis that can explain the observed relationship is that the chronicity of atopic and allergic disorders results in increased psychosocial stress. A review by Golding *et al*^16^ reported that food allergies are associated with reduced reported health-related quality of life (HRQL), and greater symptoms of depression and anxiety, related to the vigilance required to avoid exposure to triggers and associated social consequences. Furthermore, the management for atopic and allergic disorders focuses on controlling symptoms and preventing flare ups; there are no curative treatments. Repeated relapses, unsuccessful lines of treatment, and the subsequent impact on functional status represent another source of stress for individuals,^52^ which can predispose to adverse mental health. This is particularly relevant to children; many of these disorders develop in early childhood and early childhood experiences can be fundamental to the development of mental ill health and through epigenetic modifications can predispose abnormal stress responses in adults,^53^ which is a risk factor for mental health disorders. Several studies demonstrate chronic physical illness and association with mental health disorders like depression, anxiety, and ADHD.^54 55^

It is possible (indeed likely) that both of these factors contribute to the relationship between atopic disorders and mental ill health.

### Implications for clinical practice

A growing body of evidence demonstrates the impact of allergic disorders on long-term mental health outcomes, but few clinical guidelines or allergy services address this. There is a global unmet need for psychological support for patients in allergy clinics.^56 57^ A recent global survey of psychological support needs found that for adults and parents in the UK, over 80% reported psychological distress related to their or their child’s food allergy but less than 25% had been assessed for food allergy related distress and only 40% of those who needed it had been seen by a mental health professional.^56^ This pattern was similar across many other countries in Europe and North and South America. This study reinforces the growing evidence base for the integration of psychological services into standard allergy care.

### Strengths and limitations

Strengths of this study include its large and diverse sample, which is generalisable to the UK population with respect to demographics and prevalence of chronic conditions.^19 20^ To the best of our knowledge, this is the first large-scale UK primary-care based cohort study examining the relationship between atopic disorders and mental health disorders. The use of electronic health records from a primary care database reduces recall bias as data is recorded during patient consultations. The study is novel for inclusion of a range of atopic and mental health disorders- many of which are poorly studied in the existing literature.

This study has a few key limitations which must be considered when interpreting the findings. First, the study uses data from electronic healthcare records which relies on accurate documentation by healthcare professionals in general practices contributing to the dataset. Heterogeneity in recording, which can affect both the exposure and outcomes of interest, can result in misclassification bias. However, the coding of mental health disorders is more likely to be complete as SMI feature in the in the Quality and Outcomes Framework (QOF; which is a UK primary care performance management reward scheme).^58^ Additionally, while we recognised post-traumatic stress disorder as an important outcome, we could not include it in this analysis due to underdiagnosis in UK primary care settings and subsequent inconsistent coding in the database.^59^ Another limitation related to coding is that we are unable to stratify included patients by disease severity which could provide useful findings by sensitivity analysis. Similarly, factors such as educational status, parental psychiatric history which could be confounders cannot be included due to poor recording.

### Recommendations for future research

Further longitudinal studies of large community-representative samples are required to further explore the association between the broad range of atopic disorders and mental health disorders, including post-traumatic stress disorder. Future studies could explore with sub-group analyses differences in outcomes by age-of-onset and disease severity. Effect of psychological interventions, particularly on the most affected subgroups (eg, those with history of anaphylaxis), should also be measured systematically.

## Conclusion

Individuals with allergies and atopic disease were at an increased risk of developing mental ill health in this UK primary care cohort. A positive association was found between allergy and all mental health disorders (anxiety, depression, eating disorders, OCD, and self-harm) except SMI. Given the high prevalence of allergic and atopic disorders, this association underscores a substantial public mental health burden. Primary and secondary healthcare services should be aware of the potential risk of mental health disorders in patients presenting with allergic and atopic disorders. Appropriate referrals for suitable psychological support should be made where possible.

## Supplementary material

10.1136/bmjopen-2024-089181 online supplemental file 1

10.1136/bmjopen-2024-089181 online supplemental file 2

## Data Availability

Data are available upon reasonable request.
