# Supplementary material for: Association between atopic disorders and mental ill health: a UK-based retrospective cohort study
Source: BMJ Open. 2025 May 31;15(5):e089181. doi: 10.1136/bmjopen-2024-089181 (PMC12142133; doi:10.1136/bmjopen-2024-089181)
Supplement: online supplemental file 2 [file bmjopen-15-5-s002.docx]

Appendices

[Appendix B: Read Code Lists 2](#_Toc164778839)

[Table S1: Baseline Characteristics 40](#_Toc164778840)

[Table S2: Hazard ratios with 95% confidence intervals (CI) for mental ill health among patients with any atopic or allergic disorder compared to matched unexposed individuals 41](#_Toc164778841)

[Table S3: Hazard ratios with 95% confidence intervals (CI) for mental ill health among patients with food allergy only compared to matched unexposed individuals 42](#_Toc164778842)

[Table S4: Hazard ratios with 95% confidence intervals (CI) for mental ill health among patients with drug allergy only compared to matched unexposed individuals 43](#_Toc164778843)

[Table S5: Hazard ratios with 95% confidence intervals (CI) for mental ill health among patients with anaphylaxis only compared to matched unexposed individuals 44](#_Toc164778844)

[Table S6: Hazard ratios with 95% confidence intervals (CI) for mental ill health among patients with urticaria only compared to matched unexposed individuals 45](#_Toc164778845)

[Table S7: Hazard ratios with 95% confidence intervals (CI) for mental ill health among patients with allergic rhinitis only compared to matched unexposed individuals 46](#_Toc164778846)

# Appendix B: Read Code Lists

**Food Allergy**

| **Read Code** | **Description** |
| --- | --- |
| 13A6.00 | Milk free diet - allergy |
| 13A7.00 | Egg free diet - allergy |
| 14M1.00 | H/O: food allergy |
| 8CA4S00 | Dietary education for food allergy |
| 8CA4S11 | Dietary advice for food allergy |
| J432.12 | Cow's milk allergy |
| SN58.00 | Food allergy |
| SN58000 | Egg allergy |
| SN58100 | Egg protein allergy |
| SN58200 | Peanut allergy |
| SN58300 | Nut allergy |
| SN58400 | Wheat allergy |
| SN58500 | Fish allergy |
| SN58600 | Seafood allergy |
| SN58700 | Shellfish allergy |
| SN58800 | Mushroom allergy |
| SN58900 | Allergy to strawberries |
| SN58911 | Strawberry allergy |
| SN58A00 | Allergy to soya |
| SN58B00 | Allergy to banana |
| SN58C00 | Allergy to tomato |
| ZC21L00 | Advice to avoid nut intake |
| ZC2CF00 | Dietary advice for food allergy |

**Drug Allergy**

| **Read Code** | **Description** |
| --- | --- |
| 14L..00 | H/O: drug allergy |
| 14L1.00 | H/O: penicillin allergy |
| 14L2.00 | H/O: antibiotic allergy NOS |
| 14L3.00 | H/O: anaesthetic allergy |
| 14L4.00 | H/O: analgesic allergy |
| 14L5.00 | H/O: vaccine allergy |
| 14L5000 | H/O: rotavirus vaccine allergy |
| 14L6.00 | H/O: serum allergy |
| 14L7.00 | H/O: cephalosporin allergy |
| 14L8.00 | H/O: tetracycline allergy |
| 14L9.00 | H/O: gentamicin allergy |
| 14La.00 | H/O: raloxifene allergy |
| 14LA.00 | H/O: erythromycin allergy |
| 14Lb.00 | H/O: teriparatide allergy |
| 14LB.00 | H/O: neomycin allergy |
| 14Lc.00 | H/O denosumab allergy |
| 14LC.00 | H/O: chloramphenicol allergy |
| 14Ld.00 | H/O calcitonin allergy |
| 14LD.00 | H/O: sulphonamide allergy |
| 14LE.00 | H/O: trimethoprim allergy |
| 14LF.00 | H/O: co-trimoxazole allergy |
| 14LG.00 | H/O: metronidazole allergy |
| 14LH.00 | H/O: nalidixic acid allergy |
| 14LI.00 | H/O: nitrofurantoin allergy |
| 14LJ.00 | H/O: influenza vaccine allergy |
| 14LK.00 | H/O: aspirin allergy |
| 14LL.00 | H/O: betablocker allergy |
| 14LM.00 | H/O: angiotensin converting enzyme inhibitor allergy |
| 14LN.00 | H/O: angiotensin II receptor antagonist allergy |
| 14LP.00 | H/O: warfarin allergy |
| 14LQ.00 | H/O: clopidogrel allergy |
| 14LR.00 | H/O: pneumococcal vaccine allergy |
| 14LS.00 | H/O: combined calcium and vitamin D3 preparation allergy |
| 14LT.00 | H/O: bisphosphonate allergy |
| 14LT000 | H/O ibandronic acid allergy |
| 14LT200 | H/O disodium etidronate allergy |
| 14LT300 | H/O alendronic acid allergy |
| 14LT400 | H/O risedronate sodium allergy |
| 14LV.00 | H/O: selective oestrogen receptor modulator allergy |
| 14LW.00 | H/O: strontium ranelate allergy |
| 14LX.00 | H/O: dipyridamole allergy |
| 14LY.00 | Phosphodiesterase-5 inhibitor allergy |
| 14LZ.00 | H/O: drug allergy NOS |
| 1Z4..00 | Allergic reaction to drug |
| 1Z4..11 | Drug allergy |
| 1Z42.00 | Ticagrelor allergy |
| 1Z43.00 | Anticoagulant allergy |
| 1Z43000 | Rivaroxaban allergy |
| 1Z43100 | Apixaban allergy |
| SN52.12 | Allergic drug reaction NOS |
| ZV14.00 | [V]Personal history of drug allergy |
| ZV14000 | [V]Personal history of penicillin allergy |
| ZV14100 | [V]Personal history of other antibiotic allergy |
| ZV14200 | [V]Personal history of sulphonamide allergy |
| ZV14300 | [V]Personal history of other anti-infective agent allergy |
| ZV14400 | [V]Personal history of anaesthetic agent allergy |
| ZV14500 | [V]Personal history of narcotic agent allergy |
| ZV14600 | [V]Personal history of analgesic agent allergy |
| ZV14700 | [V]Personal history of serum or vaccine allergy |
| ZV14800 | [V]Personal history of aspirin allergy |
| ZV14900 | [V]Personal history of co-proxamol allergy |
| ZV14A00 | [V]Personal history of warfarin allergy |
| ZV14B00 | [V]Personal history of clopidogrel allergy |
| ZV14C00 | [V]Personal history of betablocker allergy |
| ZV14D00 | [V]PH angiotensin-converting-enzyme inhibitor allergy |
| ZV14E00 | [V]PH of angiotensin II receptor antagonist allergy |
| ZV14F00 | [V]Personal history of influenza vaccine allergy |
| ZV14G00 | [V]Personal history of pneumococcal vaccine allergy |
| ZV14H00 | [V]Personal history of strontium ranelate allergy |
| ZV14J00 | [V]PH of selective oestrogen receptor modulator allergy |
| ZV14K00 | [V]Personal history of bisphosphonate allergy |
| ZV14L00 | [V]Personal history of calcium allergy |
| ZV14M00 | [V]Personal history of vitamin D3 allergy |
| ZV14y00 | [V]Personal history of other specified drug allergy |
| ZV14z00 | [V]Personal history of unspecified drug allergy |

**Anaphylaxis**

| **Read Code** | **Description** |
| --- | --- |
| 14M5.00 | H/O: anaphylactic shock |
| SN50.00 | Anaphylactic shock |
| SN50.11 | Anaphylaxis |
| SN50000 | Anaphylactic shock due to adverse food reaction |
| SN50100 | Anaphy shock due/adv efect/correct drug or med proprly admin |
| SN59300 | Anaphylactic shock due to bee sting |
| SN59400 | Anaphylactic shock due to wasp sting |
| SP34.00 | Anaphylactic shock due to serum |
| ZV1B300 | [V]Personal history of food induced anaphylaxis |

**Urticaria**

| **Read Code** | **Description** |
| --- | --- |
| 2F8..00 | O/E - weals present |
| C373400 | Familial febrile urticaria |
| M12A200 | Solar urticaria |
| M28..00 | Urticaria |
| M280.00 | Allergic urticaria |
| M280.11 | Drug induced urticaria |
| M281.00 | Idiopathic urticaria |
| M282.00 | Urticaria due to cold and heat |
| M282000 | Cold urticaria |
| M282100 | Thermal urticaria |
| M282111 | Heat urticaria |
| M282z00 | Urticaria due to cold and heat NOS |
| M283.00 | Dermatographic urticaria |
| M283.11 | Factitial urticaria |
| M284.00 | Vibratory urticaria |
| M285.00 | Cholinergic urticaria |
| M286.00 | Contact urticaria |
| M287.00 | Physical urticaria |
| M28y.00 | Other specified urticaria |
| M28y.11 | Nettle rash |
| M28y000 | Urticaria geographica |
| M28y100 | Menstrual urticaria |
| M28y200 | Urticaria persistans |
| M28yz00 | Other specified urticaria NOS |
| M28z.00 | Urticaria NOS |
| M28z.11 | Hives |
| Myu4.00 | [X]Urticaria and erythema |
| Myu4000 | [X]Other urticaria |
| PH32100 | Urticaria pigmentosa |
| SN51.00 | Angioneurotic oedema |
| SN51.11 | Angioedema |
| SP35400 | Urticaria due to serum |

**Allergic Rhinitis**

| **Read Code** | **Description** |
| --- | --- |
| F4A3100 | Vernal conjunctivitis of limbus and cornea |
| F4C0600 | Acute atopic conjunctivitis |
| F4C0611 | Acute allergic conjunctivitis |
| F4C1300 | Vernal conjunctivitis |
| F4C1400 | Other chronic allergic conjunctivitis |
| F4C1411 | Allergic conjunctivitis |
| H17..00 | Allergic rhinitis |
| H17..11 | Perennial rhinitis |
| H17..12 | Allergic rhinosinusitis |
| H170.00 | Allergic rhinitis due to pollens |
| H170.11 | Hay fever - pollens |
| H170.12 | Pollinosis |
| H171.00 | Allergic rhinitis due to other allergens |
| H171.11 | Cat allergy |
| H171.12 | Dander (animal) allergy |
| H171.13 | Feather allergy |
| H171.14 | Hay fever - other allergen |
| H171.15 | House dust allergy |
| H171.16 | House dust mite allergy |
| H171000 | Allergy to animal |
| H171100 | Dog allergy |
| H172.00 | Allergic rhinitis due to unspecified allergen |
| H172.11 | Hay fever - unspecified allergen |
| H17z.00 | Allergic rhinitis NOS |
| H330.14 | Pollen asthma |
| Hyu2000 | [X]Other seasonal allergic rhinitis |
| Hyu2100 | [X]Other allergic rhinitis |
| SN5A.00 | Oral allergy syndrome |

**Serious mental illness**

| **Read Code** | **Description** |
| --- | --- |
| 146D.00 | H/O: manic depressive disorder |
| 1BH3.00 | Paranoid ideation |
| 1S42.00 | Manic mood |
| 212T.00 | Psychosis schizophrenia + bipolar affective disord resolved |
| 212V.00 | Bipolar affective disorder resolved |
| 225E.00 | O/E - paranoid delusions |
| 9H8..00 | On severe mental illness register |
| E1...00 | Non-organic psychoses |
| E10..00 | Schizophrenic disorders |
| E100.00 | Simple schizophrenia |
| E100.11 | Schizophrenia simplex |
| E100000 | Unspecified schizophrenia |
| E100100 | Subchronic schizophrenia |
| E100200 | Chronic schizophrenic |
| E100300 | Acute exacerbation of subchronic schizophrenia |
| E100400 | Acute exacerbation of chronic schizophrenia |
| E100500 | Schizophrenia in remission |
| E100z00 | Simple schizophrenia NOS |
| E101.00 | Hebephrenic schizophrenia |
| E101000 | Unspecified hebephrenic schizophrenia |
| E101100 | Subchronic hebephrenic schizophrenia |
| E101200 | Chronic hebephrenic schizophrenia |
| E101300 | Acute exacerbation of subchronic hebephrenic schizophrenia |
| E101400 | Acute exacerbation of chronic hebephrenic schizophrenia |
| E101500 | Hebephrenic schizophrenia in remission |
| E101z00 | Hebephrenic schizophrenia NOS |
| E102.00 | Catatonic schizophrenia |
| E102000 | Unspecified catatonic schizophrenia |
| E102100 | Subchronic catatonic schizophrenia |
| E102200 | Chronic catatonic schizophrenia |
| E102300 | Acute exacerbation of subchronic catatonic schizophrenia |
| E102400 | Acute exacerbation of chronic catatonic schizophrenia |
| E102500 | Catatonic schizophrenia in remission |
| E102z00 | Catatonic schizophrenia NOS |
| E103.00 | Paranoid schizophrenia |
| E103000 | Unspecified paranoid schizophrenia |
| E103100 | Subchronic paranoid schizophrenia |
| E103200 | Chronic paranoid schizophrenia |
| E103300 | Acute exacerbation of subchronic paranoid schizophrenia |
| E103400 | Acute exacerbation of chronic paranoid schizophrenia |
| E103500 | Paranoid schizophrenia in remission |
| E103z00 | Paranoid schizophrenia NOS |
| E104.00 | Acute schizophrenic episode |
| E105.00 | Latent schizophrenia |
| E105000 | Unspecified latent schizophrenia |
| E105100 | Subchronic latent schizophrenia |
| E105200 | Chronic latent schizophrenia |
| E105300 | Acute exacerbation of subchronic latent schizophrenia |
| E105400 | Acute exacerbation of chronic latent schizophrenia |
| E105500 | Latent schizophrenia in remission |
| E105z00 | Latent schizophrenia NOS |
| E106.00 | Residual schizophrenia |
| E106.11 | Restzustand - schizophrenia |
| E107.00 | Schizo-affective schizophrenia |
| E107.11 | Cyclic schizophrenia |
| E107000 | Unspecified schizo-affective schizophrenia |
| E107100 | Subchronic schizo-affective schizophrenia |
| E107200 | Chronic schizo-affective schizophrenia |
| E107300 | Acute exacerbation subchronic schizo-affective schizophrenia |
| E107400 | Acute exacerbation of chronic schizo-affective schizophrenia |
| E107500 | Schizo-affective schizophrenia in remission |
| E107z00 | Schizo-affective schizophrenia NOS |
| E10y.00 | Other schizophrenia |
| E10y.11 | Cenesthopathic schizophrenia |
| E10y000 | Atypical schizophrenia |
| E10y100 | Coenesthopathic schizophrenia |
| E10yz00 | Other schizophrenia NOS |
| E10z.00 | Schizophrenia NOS |
| E11..11 | Bipolar psychoses |
| E11..13 | Manic psychoses |
| E110.00 | Manic disorder single episode |
| E110.11 | Hypomanic psychoses |
| E110000 | Single manic episode unspecified |
| E110100 | Single manic episode mild |
| E110200 | Single manic episode moderate |
| E110300 | Single manic episode severe without mention of psychosis |
| E110400 | Single manic episode severe with psychosis |
| E110500 | Single manic episode in partial or unspecified remission |
| E110600 | Single manic episode in full remission |
| E110z00 | Manic disorder single episode NOS |
| E111.00 | Recurrent manic episodes |
| E111000 | Recurrent manic episodes unspecified |
| E111100 | Recurrent manic episodes mild |
| E111200 | Recurrent manic episodes moderate |
| E111300 | Recurrent manic episodes severe without mention psychosis |
| E111400 | Recurrent manic episodes severe with psychosis |
| E111500 | Recurrent manic episodes partial or unspecified remission |
| E111600 | Recurrent manic episodes in full remission |
| E111z00 | Recurrent manic episode NOS |
| E114.00 | Bipolar affective disorder currently manic |
| E114.11 | Manic-depressive - now manic |
| E114000 | Bipolar affective disorder currently manic unspecified |
| E114100 | Bipolar affective disorder currently manic mild |
| E114200 | Bipolar affective disorder currently manic moderate |
| E114300 | Bipolar affect disord currently manic severe no psychosis |
| E114400 | Bipolar affect disord currently manic severe with psychosis |
| E114500 | Bipolar affect disord currently manic part/unspec remission |
| E114600 | Bipolar affective disorder currently manic full remission |
| E114z00 | Bipolar affective disorder currently manic NOS |
| E115.00 | Bipolar affective disorder currently depressed |
| E115.11 | Manic-depressive - now depressed |
| E115000 | Bipolar affective disorder currently depressed unspecified |
| E115100 | Bipolar affective disorder currently depressed mild |
| E115200 | Bipolar affective disorder currently depressed moderate |
| E115300 | Bipolar affect disord now depressed severe no psychosis |
| E115400 | Bipolar affect disord now depressed severe with psychosis |
| E115500 | Bipolar affect disord now depressed part/unspec remission |
| E115600 | Bipolar affective disorder now depressed in full remission |
| E115z00 | Bipolar affective disorder currently depressed NOS |
| E116.00 | Mixed bipolar affective disorder |
| E116000 | Mixed bipolar affective disorder unspecified |
| E116100 | Mixed bipolar affective disorder mild |
| E116200 | Mixed bipolar affective disorder moderate |
| E116300 | Mixed bipolar affective disorder severe no psychosis |
| E116400 | Mixed bipolar affective disorder severe with psychosis |
| E116500 | Mixed bipolar affective disorder partial/unspec remission |
| E116600 | Mixed bipolar affective disorder in full remission |
| E116z00 | Mixed bipolar affective disorder NOS |
| E117.00 | Unspecified bipolar affective disorder |
| E117000 | Unspecified bipolar affective disorder unspecified |
| E117100 | Unspecified bipolar affective disorder mild |
| E117200 | Unspecified bipolar affective disorder moderate |
| E117300 | Unspecified bipolar affective disorder severe no psychosis |
| E117400 | Unspecified bipolar affective disorder severe with psychosis |
| E117500 | Unspecified bipolar affect disord partial/unspec remission |
| E117600 | Unspecified bipolar affective disorder in full remission |
| E117z00 | Unspecified bipolar affective disorder NOS |
| E11y.00 | Other and unspecified manic-depressive psychoses |
| E11y000 | Unspecified manic-depressive psychoses |
| E11y100 | Atypical manic disorder |
| E11y300 | Other mixed manic-depressive psychoses |
| E11yz00 | Other and unspecified manic-depressive psychoses NOS |
| E12..00 | Paranoid states |
| E120.00 | Simple paranoid state |
| E121.00 | Chronic paranoid psychosis |
| E123.00 | Shared paranoid disorder |
| E12y.00 | Other paranoid states |
| E12yz00 | Other paranoid states NOS |
| E12z.00 | Paranoid psychosis NOS |
| E13..00 | Other nonorganic psychoses |
| E13..11 | Reactive psychoses |
| E131.00 | Acute hysterical psychosis |
| E133.00 | Acute paranoid reaction |
| E134.00 | Psychogenic paranoid psychosis |
| E13y.00 | Other reactive psychoses |
| E13y000 | Psychogenic stupor |
| E13y100 | Brief reactive psychosis |
| E13yz00 | Other reactive psychoses NOS |
| E13z.00 | Nonorganic psychosis NOS |
| E13z.11 | Psychotic episode NOS |
| E14..00 | Psychoses with origin in childhood |
| E141.00 | Disintegrative psychosis |
| E14y.00 | Other childhood psychoses |
| E14y000 | Atypical childhood psychoses |
| E14y100 | Borderline psychosis of childhood |
| E14yz00 | Other childhood psychoses NOS |
| E14z.00 | Child psychosis NOS |
| E14z.11 | Childhood schizophrenia NOS |
| E1y..00 | Other specified non-organic psychoses |
| E1z..00 | Non-organic psychosis NOS |
| Eu2..00 | [X]Schizophrenia schizotypal and delusional disorders |
| Eu20.00 | [X]Schizophrenia |
| Eu20000 | [X]Paranoid schizophrenia |
| Eu20011 | [X]Paraphrenic schizophrenia |
| Eu20100 | [X]Hebephrenic schizophrenia |
| Eu20111 | [X]Disorganised schizophrenia |
| Eu20200 | [X]Catatonic schizophrenia |
| Eu20211 | [X]Catatonic stupor |
| Eu20212 | [X]Schizophrenic catalepsy |
| Eu20213 | [X]Schizophrenic catatonia |
| Eu20214 | [X]Schizophrenic flexibilatis cerea |
| Eu20300 | [X]Undifferentiated schizophrenia |
| Eu20311 | [X]Atypical schizophrenia |
| Eu20400 | [X]Post-schizophrenic depression |
| Eu20500 | [X]Residual schizophrenia |
| Eu20511 | [X]Chronic undifferentiated schizophrenia |
| Eu20512 | [X]Restzustand schizophrenic |
| Eu20600 | [X]Simple schizophrenia |
| Eu20y00 | [X]Other schizophrenia |
| Eu20y11 | [X]Cenesthopathic schizophrenia |
| Eu20y12 | [X]Schizophreniform disord NOS |
| Eu20y13 | [X]Schizophrenifrm psychos NOS |
| Eu20z00 | [X]Schizophrenia unspeified |
| Eu21.00 | [X]Schizotypal disorder |
| Eu21.11 | [X]Latent schizophrenic reaction |
| Eu21.12 | [X]Borderline schizophrenia |
| Eu21.13 | [X]Latent schizophrenia |
| Eu21.14 | [X]Prepsychotic schizophrenia |
| Eu21.15 | [X]Prodromal schizophrenia |
| Eu21.16 | [X]Pseudoneurotic schizophrenia |
| Eu21.17 | [X]Pseudopsychopathic schizophrenia |
| Eu22.00 | [X]Persistent delusional disorders |
| Eu22000 | [X]Delusional disorder |
| Eu22011 | [X]Paranoid psychosis |
| Eu22012 | [X]Paranoid state |
| Eu22013 | [X]Paraphrenia - late |
| Eu22014 | [X]Sensitiver Beziehungswahn |
| Eu22015 | [X]Paranoia |
| Eu22300 | [X]Paranoid state in remission |
| Eu22y12 | [X]Involutional paranoid state |
| Eu23012 | [X]Cycloid psychosis |
| Eu23100 | [X]Acute polymorphic psychot disord with symp of schizophren |
| Eu23111 | [X]Bouffee delirante with symptoms of schizophrenia |
| Eu23112 | [X]Cycloid psychosis with symptoms of schizophrenia |
| Eu23200 | [X]Acute schizophrenia-like psychotic disorder |
| Eu23211 | [X]Brief schizophreniform disorder |
| Eu23212 | [X]Brief schizophrenifrm psych |
| Eu23214 | [X]Schizophrenic reaction |
| Eu23312 | [X]Psychogenic paranoid psychosis |
| Eu23y00 | [X]Other acute and transient psychotic disorders |
| Eu23z00 | [X]Acute and transient psychotic disorder unspecified |
| Eu23z11 | [X]Brief reactive psychosis NOS |
| Eu23z12 | [X]Reactive psychosis |
| Eu24.12 | [X]Induced paranoid disorder |
| Eu25.00 | [X]Schizoaffective disorders |
| Eu25000 | [X]Schizoaffective disorder manic type |
| Eu25011 | [X]Schizoaffective psychosis manic type |
| Eu25012 | [X]Schizophreniform psychosis manic type |
| Eu25100 | [X]Schizoaffective disorder depressive type |
| Eu25111 | [X]Schizoaffective psychosis depressive type |
| Eu25112 | [X]Schizophreniform psychosis depressive type |
| Eu25200 | [X]Schizoaffective disorder mixed type |
| Eu25211 | [X]Cyclic schizophrenia |
| Eu25212 | [X]Mixed schizophrenic and affective psychosis |
| Eu25y00 | [X]Other schizoaffective disorders |
| Eu25z00 | [X]Schizoaffective disorder unspecified |
| Eu25z11 | [X]Schizoaffective psychosis NOS |
| Eu26.00 | [X]Nonorganic psychosis in remission |
| Eu2y.00 | [X]Other nonorganic psychotic disorders |
| Eu2y.11 | [X]Chronic hallucinatory psychosis |
| Eu2z.00 | [X]Unspecified nonorganic psychosis |
| Eu2z.11 | [X]Psychosis NOS |
| Eu30.00 | [X]Manic episode |
| Eu30.11 | [X]Bipolar disorder single manic episode |
| Eu30000 | [X]Hypomania |
| Eu30100 | [X]Mania without psychotic symptoms |
| Eu30200 | [X]Mania with psychotic symptoms |
| Eu30211 | [X]Mania with mood-congruent psychotic symptoms |
| Eu30212 | [X]Mania with mood-incongruent psychotic symptoms |
| Eu30213 | [X]Manic stupor |
| Eu30y00 | [X]Other manic episodes |
| Eu30z00 | [X]Manic episode unspecified |
| Eu30z11 | [X]Mania NOS |
| Eu31.00 | [X]Bipolar affective disorder |
| Eu31.11 | [X]Manic-depressive illness |
| Eu31.12 | [X]Manic-depressive psychosis |
| Eu31.13 | [X]Manic-depressive reaction |
| Eu31000 | [X]Bipolar affective disorder current episode hypomanic |
| Eu31100 | [X]Bipolar affect disorder cur epi manic wout psychotic symp |
| Eu31200 | [X]Bipolar affect disorder cur epi manic with psychotic symp |
| Eu31300 | [X]Bipolar affect disorder cur epi mild or moderate depressn |
| Eu31400 | [X]Bipol aff disord curr epis sev depress no psychot symp |
| Eu31500 | [X]Bipolar affect dis cur epi severe depres with psyc symp |
| Eu31600 | [X]Bipolar affective disorder current episode mixed |
| Eu31700 | [X]Bipolar affective disorder currently in remission |
| Eu31800 | [X]Bipolar affective disorder type I |
| Eu31900 | [X]Bipolar affective disorder type II |
| Eu31911 | [X]Bipolar II disorder |
| Eu31y00 | [X]Other bipolar affective disorders |
| Eu31y11 | [X]Bipolar II disorder |
| Eu31y12 | [X]Recurrent manic episodes |
| Eu31z00 | [X]Bipolar affective disorder unspecified |
| Eu33213 | [X]Manic-depress psychosis depressd no psychotic symptoms |
| Eu33312 | [X]Manic-depress psychosis depressed type+psychotic symptoms |
| ZRby100 | Profile of mood states bipolar |
| ZV11111 | [V]Personal history of manic-depressive psychosis |
| ZV11112 | [V]Personal history of manic-depressive psychosis |

**Anxiety**

| **Read Code** | **Description** |
| --- | --- |
| 146G.00 | H/O: agoraphobia |
| 8CAZ000 | Patient given advice about management of anxiety |
| 8HHp.00 | Referral for guided self-help for anxiety |
| E20..00 | Neurotic disorders |
| E200.00 | Anxiety states |
| E200000 | Anxiety state unspecified |
| E200100 | Panic disorder |
| E200200 | Generalised anxiety disorder |
| E200400 | Chronic anxiety |
| E200500 | Recurrent anxiety |
| E200z00 | Anxiety state NOS |
| E202.00 | Phobic disorders |
| E202.11 | Social phobic disorders |
| E202.12 | Phobic anxiety |
| E202000 | Phobia unspecified |
| E202100 | Agoraphobia with panic attacks |
| E202200 | Agoraphobia without mention of panic attacks |
| E202300 | Social phobia fear of eating in public |
| E202400 | Social phobia fear of public speaking |
| E202500 | Social phobia fear of public washing |
| E202600 | Acrophobia |
| E202700 | Animal phobia |
| E202800 | Claustrophobia |
| E202900 | Fear of crowds |
| E202B00 | Cancer phobia |
| E202C00 | Dental phobia |
| E202E00 | Fear of pregnancy |
| E202z00 | Phobic disorder NOS |
| E20y.00 | Other neurotic disorders |
| E20y200 | Other occupational neurosis |
| E20y300 | Psychasthenic neurosis |
| E20yz00 | Other neurotic disorder NOS |
| E20z.00 | Neurotic disorder NOS |
| E28..00 | Acute reaction to stress |
| E280.00 | Acute panic state due to acute stress reaction |
| E281.00 | Acute fugue state due to acute stress reaction |
| E282.00 | Acute stupor state due to acute stress reaction |
| E283.00 | Other acute stress reactions |
| E283100 | Acute posttrauma stress state |
| E283z00 | Other acute stress reaction NOS |
| E284.00 | Stress reaction causing mixed disturbance of emotion/conduct |
| E28z.00 | Acute stress reaction NOS |
| E28z.12 | Flying phobia |
| Eu22y11 | [X]Delusional dysmorphophobia |
| Eu4..00 | [X]Neurotic stress - related and somoform disorders |
| Eu40.00 | [X]Phobic anxiety disorders |
| Eu40000 | [X]Agoraphobia |
| Eu40011 | [X]Agoraphobia without history of panic disorder |
| Eu40012 | [X]Panic disorder with agoraphobia |
| Eu40100 | [X]Social phobias |
| Eu40111 | [X]Anthropophobia |
| Eu40112 | [X]Social neurosis |
| Eu40200 | [X]Specific (isolated) phobias |
| Eu40211 | [X]Acrophobia |
| Eu40212 | [X]Animal phobias |
| Eu40213 | [X]Claustrophobia |
| Eu40214 | [X]Simple phobia |
| Eu40300 | [X]Needle phobia |
| Eu40y00 | [X]Other phobic anxiety disorders |
| Eu40z00 | [X]Phobic anxiety disorder unspecified |
| Eu40z11 | [X]Phobia NOS |
| Eu40z12 | [X]Phobic state NOS |
| Eu41.00 | [X]Other anxiety disorders |
| Eu41000 | [X]Panic disorder [episodic paroxysmal anxiety] |
| Eu41100 | [X]Generalized anxiety disorder |
| Eu41111 | [X]Anxiety neurosis |
| Eu41112 | [X]Anxiety reaction |
| Eu41113 | [X]Anxiety state |
| Eu41300 | [X]Other mixed anxiety disorders |
| Eu41y00 | [X]Other specified anxiety disorders |
| Eu41y11 | [X]Anxiety hysteria |
| Eu41z00 | [X]Anxiety disorder unspecified |
| Eu41z11 | [X]Anxiety NOS |
| Eu42.11 | [X]Anankastic neurosis |
| Eu42.12 | [X]Obsessive-compulsive neurosis |
| Eu43.00 | [X]Reaction to severe stress and adjustment disorders |
| Eu43000 | [X]Acute stress reaction |
| Eu43012 | [X]Acute reaction to stress |
| Eu43y00 | [X]Other reactions to severe stress |
| Eu43z00 | [X]Reaction to severe stress unspecified |
| Eu45212 | [X]Dysmorphophobia nondelusional |
| Eu45215 | [X]Nosophobia |
| Eu51511 | [X]Dream anxiety disorder |
| Z481.00 | Phobia counselling |
| Z4L1.00 | Anxiety counselling |
| Z522400 | Desensitisation - phobia |
| Z522600 | Flooding - obsessional compulsive disorder |
| Z522700 | Flooding - agoraphobia |

**Depression**

| **Read Code** | **Description** |
| --- | --- |
| E112.00 | Single major depressive episode |
| E112.11 | Agitated depression |
| E112.12 | Endogenous depression first episode |
| E112.13 | Endogenous depression first episode |
| E112.14 | Endogenous depression |
| E112000 | Single major depressive episode unspecified |
| E112100 | Single major depressive episode mild |
| E112200 | Single major depressive episode moderate |
| E112300 | Single major depressive episode severe without psychosis |
| E112400 | Single major depressive episode severe with psychosis |
| E112500 | Single major depressive episode partial or unspec remission |
| E112600 | Single major depressive episode in full remission |
| E112z00 | Single major depressive episode NOS |
| E113.00 | Recurrent major depressive episode |
| E113.11 | Endogenous depression - recurrent |
| E113000 | Recurrent major depressive episodes unspecified |
| E113100 | Recurrent major depressive episodes mild |
| E113200 | Recurrent major depressive episodes moderate |
| E113300 | Recurrent major depressive episodes severe no psychosis |
| E113400 | Recurrent major depressive episodes with psychosis |
| E113500 | Recurrent major depressive episodes partial/unspec remission |
| E113600 | Recurrent major depressive episodes in full remission |
| E113700 | Recurrent depression |
| E113z00 | Recurrent major depressive episode NOS |
| E118.00 | Seasonal affective disorder |
| E11y200 | Atypical depressive disorder |
| E11z200 | Masked depression |
| E130.00 | Reactive depressive psychosis |
| E135.00 | Agitated depression |
| E291.00 | Prolonged depressive reaction |
| E2B..00 | Depressive disorder NEC |
| E2B1.00 | Chronic depression |
| Eu32.00 | [X]Depressive episode |
| Eu32.11 | [X]Single episode of depressive reaction |
| Eu32.12 | [X]Single episode of psychogenic depression |
| Eu32.13 | [X]Single episode of reactive depression |
| Eu32000 | [X]Mild depressive episode |
| Eu32100 | [X]Moderate depressive episode |
| Eu32200 | [X]Severe depressive episode without psychotic symptoms |
| Eu32211 | [X]Single episode agitated depressn w'out psychotic symptoms |
| Eu32212 | [X]Single episode major depression w'out psychotic symptoms |
| Eu32213 | [X]Single episode vital depression w'out psychotic symptoms |
| Eu32300 | [X]Severe depressive episode with psychotic symptoms |
| Eu32311 | [X]Single episode of major depression and psychotic symptoms |
| Eu32312 | [X]Single episode of psychogenic depressive psychosis |
| Eu32313 | [X]Single episode of psychotic depression |
| Eu32314 | [X]Single episode of reactive depressive psychosis |
| Eu32400 | [X]Mild depression |
| Eu32500 | [X]Major depression mild |
| Eu32600 | [X]Major depression moderately severe |
| Eu32700 | [X]Major depression severe without psychotic symptoms |
| Eu32800 | [X]Major depression severe with psychotic symptoms |
| Eu32y00 | [X]Other depressive episodes |
| Eu32y11 | [X]Atypical depression |
| Eu32y12 | [X]Single episode of masked depression NOS |
| Eu32z00 | [X]Depressive episode unspecified |
| Eu32z11 | [X]Depression NOS |
| Eu32z12 | [X]Depressive disorder NOS |
| Eu32z13 | [X]Prolonged single episode of reactive depression |
| Eu32z14 | [X] Reactive depression NOS |
| Eu33.00 | [X]Recurrent depressive disorder |
| Eu33.11 | [X]Recurrent episodes of depressive reaction |
| Eu33.12 | [X]Recurrent episodes of psychogenic depression |
| Eu33.13 | [X]Recurrent episodes of reactive depression |
| Eu33.14 | [X]Seasonal depressive disorder |
| Eu33.15 | [X]SAD - Seasonal affective disorder |
| Eu33000 | [X]Recurrent depressive disorder current episode mild |
| Eu33100 | [X]Recurrent depressive disorder current episode moderate |
| Eu33200 | [X]Recurr depress disorder cur epi severe without psyc sympt |
| Eu33211 | [X]Endogenous depression without psychotic symptoms |
| Eu33212 | [X]Major depression recurrent without psychotic symptoms |
| Eu33214 | [X]Vital depression recurrent without psychotic symptoms |
| Eu33300 | [X]Recurrent depress disorder cur epi severe with psyc symp |
| Eu33311 | [X]Endogenous depression with psychotic symptoms |
| Eu33313 | [X]Recurr severe episodes/major depression+psychotic symptom |
| Eu33314 | [X]Recurr severe episodes/psychogenic depressive psychosis |
| Eu33315 | [X]Recurrent severe episodes of psychotic depression |
| Eu33316 | [X]Recurrent severe episodes/reactive depressive psychosis |
| Eu33400 | [X]Recurrent depressive disorder currently in remission |
| Eu33y00 | [X]Other recurrent depressive disorders |
| Eu33z00 | [X]Recurrent depressive disorder unspecified |
| Eu33z11 | [X]Monopolar depression NOS |
| Eu34100 | [X]Dysthymia |

**Eating Disorders**

| **Read Code** | **Description** |
| --- | --- |
| 1467.00 | H/O: anorexia nervosa |
| 1JZ..00 | Suspected binge eating disorder |
| 8HTN.00 | Referral to eating disorders clinic |
| 9Nk9.00 | Seen in eating disorder clinic |
| E275200 | Pica |
| E264200 | Cyclical vomiting - psychogenic |
| E271.00 | Anorexia nervosa |
| E275.00 | Other and unspecified non-organic eating disorders |
| E275000 | Unspecified non-organic eating disorder |
| E275100 | Bulimia (non-organic overeating) |
| E275111 | Compulsive eating disorder |
| E275400 | Psychogenic vomiting NOS |
| E275y00 | Other specified non-organic eating disorder |
| E275z00 | Non-organic eating disorder NOS |
| Eu50.00 | [X]Eating disorders |
| Eu50000 | [X]Anorexia nervosa |
| Eu50100 | [X]Atypical anorexia nervosa |
| Eu50200 | [X]Bulimia nervosa |
| Eu50211 | [X]Bulimia NOS |
| Eu50212 | [X]Hyperorexia nervosa |
| Eu50300 | [X]Atypical bulimia nervosa |
| Eu50400 | [X]Overeating associated with other psychological disturbncs |
| Eu50411 | [X]Psychogenic overeating |
| Eu50500 | [X]Vomiting associated with other psychological disturbances |
| Eu50511 | [X]Psychogenic vomiting |
| Eu50y00 | [X]Other eating disorders |
| Eu50y12 | [X]Psychogenic loss of appetite |
| Eu50z00 | [X]Eating disorder |
| Fy05.00 | Nocturnal sleep-related eating disorder |
| R036011 | [D]Bulimia NOS |
| Z4B5.00 | Eating disorder counselling |
| ZC2CD00 | Dietary advice for eating disorder |

**Obsessive Compulsive Disorder (OCD)**

| **Read Code** | **Description** |
| --- | --- |
| E203.00 | Obsessive-compulsive disorders |
| E203.11 | Anancastic neurosis |
| E203000 | Compulsive neurosis |
| E203100 | Obsessional neurosis |
| E203z00 | Obsessive-compulsive disorder NOS |
| Eu42.00 | [X]Obsessive - compulsive disorder |
| Eu42.11 | [X]Anankastic neurosis |
| Eu42.12 | [X]Obsessive-compulsive neurosis |
| Eu42000 | [X]Predominantly obsessional thoughts or ruminations |
| Eu42100 | [X]Predominantly compulsive acts [obsessional rituals] |
| Eu42200 | [X]Mixed obsessional thoughts and acts |
| Eu42y00 | [X]Other obsessive-compulsive disorders |
| Eu42z00 | [X]Obsessive-compulsive disorder unspecified |

**Self-harm**

| **Read Code** | **Description** |
| --- | --- |
| ZX1H.00 | Self-asphyxiation |
| 146B.00 | H/O: deliberate self harm |
| 146A.00 | H/O: attempted suicide |
| 14K1.00 | Intentional overdose of prescription only medication |
| 14K0.00 | H/O: repeated overdose |
| 1BD4.00 | Suicide risk |
| 1BD8.00 | At risk of DSH - deliberate self harm |
| 1BDA.00 | Thoughts of deliberate self harm |
| 1BD6.00 | Moderate suicide risk |
| 1BDC.00 | Intent of deliberate self harm with detailed plans |
| 1BDB.00 | Plans for deliberate self harm without intent |
| 1JP..00 | Suspected drug overdose |
| 8G6..00 | Anti-suicide psychotherapy |
| SL...14 | Overdose of biological substance |
| SL90.00 | Antidepressant poisoning |
| SL90z00 | Anti-depressant poisoning NOS |
| TK...17 | Para-suicide |
| TK01011 | Suicide and self inflicted injury by amobarbital |
| TK01200 | Suicide and self inflicted injury by Butabarbitone |
| TK01411 | Suicide and self inflicted injury by phenobarbital |
| TK01500 | Suicide and self inflicted injury by Quinalbarbitone |
| TK01511 | Suicide and self inflicted injury by secobarbital |
| TK55.00 | Suicide and selfinflicted injury by explosives |
| TK...13 | Poisoning - self-inflicted |
| TK60100 | Self inflicted lacerations to wrist |
| TK00.00 | Suicide + selfinflicted poisoning by analgesic/antipyretic |
| TK...12 | Injury - self-inflicted |
| TK0..00 | Suicide + selfinflicted poisoning by solid/liquid substances |
| TK04.00 | Suicide + selfinflicted poisoning by other drugs/medicines |
| TK60111 | Slashed wrists self inflicted |
| TK30.00 | Suicide and selfinflicted injury by hanging |
| TK...14 | Suicide and self harm |
| TK60.00 | Suicide and selfinflicted injury by cutting |
| TK03.00 | Suicide + selfinflicted poisoning tranquilliser/psychotropic |
| TK02.00 | Suicide + selfinflicted poisoning by oth sedatives/hypnotics |
| TK3..00 | Suicide + selfinflicted injury by hang/strangulate/suffocate |
| TK6..00 | Suicide and selfinflicted injury by cutting and stabbing |
| TK0z.00 | Suicide + selfinflicted poisoning by solid/liquid subst NOS |
| TK...11 | Cause of overdose - deliberate |
| TK01.00 | Suicide + selfinflicted poisoning by barbiturates |
| TK61.00 | Suicide and selfinflicted injury by stabbing |
| TK7..00 | Suicide and selfinflicted injury by jumping from high place |
| TK4..00 | Suicide and selfinflicted injury by drowning |
| TK...15 | Attempted suicide |
| TK3y.00 | Suicide + selfinflicted inj oth mean hang/strangle/suffocate |
| TK6z.00 | Suicide and selfinflicted injury by cutting and stabbing NOS |
| TKx..00 | Suicide and selfinflicted injury by other means |
| TK1..00 | Suicide + selfinflicted poisoning by gases in domestic use |
| TK07.00 | Suicide + selfinflicted poisoning by corrosive/caustic subst |
| TK51.00 | Suicide and selfinflicted injury by shotgun |
| TK7z.00 | Suicide+selfinflicted injury-jump from high place NOS |
| TK2..00 | Suicide + selfinflicted poisoning by other gases and vapours |
| TK21.00 | Suicide and selfinflicted poisoning by other carbon monoxide |
| TK70.00 | Suicide+selfinflicted injury-jump from residential premises |
| TK3z.00 | Suicide + selfinflicted inj by hang/strangle/suffocate NOS |
| TK01400 | Suicide and self inflicted injury by Phenobarbitone |
| TK71.00 | Suicide+selfinflicted injury-jump from oth manmade structure |
| TK1z.00 | Suicide + selfinflicted poisoning by domestic gases NOS |
| TK31.00 | Suicide + selfinflicted injury by suffocation by plastic bag |
| TK5..00 | Suicide and selfinflicted injury by firearms and explosives |
| TKx0.00 | Suicide + selfinflicted injury-jump/lie before moving object |
| TK06.00 | Suicide + selfinflicted poisoning by agricultural chemical |
| TK01z00 | Suicide and self inflicted injury by barbiturates |
| TK10.00 | Suicide + selfinflicted poisoning by gas via pipeline |
| TK72.00 | Suicide+selfinflicted injury-jump from natural sites |
| TK11.00 | Suicide + selfinflicted poisoning by liquified petrol gas |
| TK2z.00 | Suicide + selfinflicted poisoning by gases and vapours NOS |
| TK01000 | Suicide and self inflicted injury by Amylobarbitone |
| TK2y.00 | Suicide + selfinflicted poisoning by other gases and vapours |
| TK52.00 | Suicide and selfinflicted injury by hunting rifle |
| TK53.00 | Suicide and selfinflicted injury by military firearms |
| TK5z.00 | Suicide and selfinflicted injury by firearms/explosives NOS |
| TK01100 | Suicide and self inflicted injury by Barbitone |
| TK01300 | Suicide and self inflicted injury by Pentabarbitone |
| TK08.00 | Suicide + selfinflicted poisoning by arsenic + its compounds |
| TK1y.00 | Suicide and selfinflicted poisoning by other utility gas |
| TK50.00 | Suicide and selfinflicted injury by handgun |
| TK...00 | Suicide and selfinflicted injury |
| TKx0z00 | Suicide + selfinflicted inj-jump/lie before moving obj NOS |
| TKz..00 | Suicide and selfinflicted injury NOS |
| TKx1.00 | Suicide and selfinflicted injury by burns or fire |
| TKx2.00 | Suicide and selfinflicted injury by scald |
| TKy..00 | Late effects of selfinflicted injury |
| TKxz.00 | Suicide and selfinflicted injury by other means NOS |
| TKxy.00 | Suicide and selfinflicted injury by other specified means |
| TKx0000 | Suicide + selfinflicted injury-jumping before moving object |
| TKx4.00 | Suicide and selfinflicted injury by electrocution |
| TKx5.00 | Suicide and selfinflicted injury by crashing motor vehicle |
| TKx6.00 | Suicide and selfinflicted injury by crashing of aircraft |
| TKx3.00 | Suicide and selfinflicted injury by extremes of cold |
| U200100 | [X]Intent self poison nonopioid analgesic at res institut |
| U200300 | [X]Int self poison nonopioid analges in sport/athletic area |
| U200400 | [X]Intent self pois nonopioid analgesic in street/highway |
| U200700 | [X]Int self poison/exposure to nonopioid analgesic on farm |
| U201100 | [X]Intent self poison antiepileptic at res institut |
| U201200 | [X]Intent self pois nonopioid analges school/pub admin area |
| U201300 | [X]Int self poison antiepileptic in sport/athletic area |
| U201400 | [X]Intent self pois antiepileptic in street/highway |
| U201500 | [X]Intent self pois antiepileptic trade/service area |
| U201600 | [X]Int self poison antiepileptic indust/construct area |
| U201700 | [X]Int self poison/exposure to antiepileptic on farm |
| U201y00 | [X]Intent self poison antiepileptic other spec place |
| U202100 | [X]Intent self poison sedative hypnotic at res institut |
| U202200 | [X]Int self poison sedative hypnotic school/pub admin area |
| U202300 | [X]Int self poison sedative hypnotic in sport/athletic area |
| U2...11 | [X]Self inflicted injury |
| U2...14 | [X]Attempted suicide |
| U2...15 | [X]Para-suicide |
| U2...13 | [X]Suicide |
| U200.00 | [X]Intent self poison/exposure to nonopioid analgesic |
| U2...12 | [X]Injury - self-inflicted |
| U200.13 | [X]Overdose - aspirin |
| U202.12 | [X]Overdose - diazepam |
| U202.00 | [X]Intent self poison/exposure to sedative hypnotic |
| U2...00 | [X]Intentional self-harm |
| U202.16 | [X]Overdose - benzodiazepine |
| U200.12 | [X]Overdose - ibuprofen |
| U202.11 | [X]Overdose - sleeping tabs |
| U202.13 | [X]Overdose - temazepam |
| U20..00 | [X]Intentional self poisoning/exposure to noxious substances |
| U200000 | [X]Int self poison/exposure to nonopioid analgesic at home |
| U202.17 | [X]Overdose - barbiturate |
| U200z00 | [X]Intent self poison nonopioid analgesic unspecif place |
| U201.00 | [X]Intent self poison/exposure to antiepileptic |
| U202.15 | [X]Overdose - nitrazepam |
| U202000 | [X]Int self poison/exposure to sedative hypnotic at home |
| U201z00 | [X]Intent self poison antiepileptic unspecif place |
| U201000 | [X]Int self poison/exposure to antiepileptic at home |
| U200600 | [X]Int self pois nonopioid analgesic indust/construct area |
| U200y00 | [X]Int self poison nonopioid analgesic other spec place |
| U202.18 | [X]Overdose - amobarbital |
| U200200 | [X]Int self poison nonopioid analges school/pub admin area |
| U202.14 | [X]Overdose - flurazepam |
| U200.11 | [X]Overdose - paracetamol |
| U200500 | [X]Intent self pois nonopioid analgesic trade/service area |
| U202400 | [X]Intent self pois sedative hypnotic in street/highway |
| U202500 | [X]Intent self pois sedative hypnotic trade/service area |
| U202600 | [X]Int self pois sedative hypnotic indust/construct area |
| U202700 | [X]Int self poison/exposure to sedative hypnotic on farm |
| U203000 | [X]Int self poison/exposure to antiparkinson drug at home |
| U203100 | [X]Intent self poison antiparkinson drug at res institut |
| U203200 | [X]Int self poison antparkinson drug school/pub admin area |
| U203300 | [X]Int self poison antparkinson drug in sport/athletic area |
| U203400 | [X]Intent self pois antiparkinson drug in street/highway |
| U203500 | [X]Intent self pois antiparkinson drug trade/service area |
| U203600 | [X]Int self pois antiparkinson drug indust/construct area |
| U203700 | [X]Int self poison/exposure to antiparkinson drug on farm |
| U203y00 | [X]Int self poison antiparkinson drug other spec place |
| U203z00 | [X]Intent self poison antiparkinson drug unspecif place |
| U204100 | [X]Intent self poison psychotropic drug at res institut |
| U204200 | [X]Int self poison psychotropic drug school/pub admin area |
| U204300 | [X]Int self poison psychotropic drug in sport/athletic area |
| U204400 | [X]Intent self pois psychotropic drug in street/highway |
| U204500 | [X]Intent self pois psychotropic drug trade/service area |
| U204600 | [X]Int self pois psychotropic drug indust/construct area |
| U204700 | [X]Int self poison/exposure to psychotropic drug on farm |
| U205100 | [X]Intent self poison narcotic drug at res institut |
| U205200 | [X]Int self poison narcotic drug school/pub admin area |
| U205300 | [X]Int self poison narcotic drug in sport/athletic area |
| U205400 | [X]Intent self pois narcotic drug in street/highway |
| U205500 | [X]Intent self pois narcotic drug trade/service area |
| U205600 | [X]Int self pois narcotic drug indust/construct area |
| U205700 | [X]Int self poison/exposure to narcotic drug on farm |
| U206100 | [X]Intent self poison hallucinogen at res institut |
| U206200 | [X]Int self poison hallucinogenschool/pub admin area |
| U206300 | [X]Int self poison hallucinogenin sport/athletic area |
| U206400 | [X]Intent self pois hallucinogen in street/highway |
| U206500 | [X]Intent self pois hallucinogen trade/service area |
| U206600 | [X]Int self pois hallucinogen indust/construct area |
| U206700 | [X]Int self poison/exposure to hallucinogen on farm |
| U206y00 | [X]Int self poison hallucinogen other spec place |
| U207100 | [X]Intent self poison oth autonomic drug at res institut |
| U207200 | [X]Int self poison oth autonom drug school/pub admin area |
| U207300 | [X]Int self poison oth autonom drug in sport/athletic area |
| U207400 | [X]Intent self pois oth autonomic drug in street/highway |
| U207500 | [X]Intent self pois oth autonomic drug trade/service area |
| U207600 | [X]Int self pois oth autonomic drug indust/construct area |
| U207700 | [X]Int self poison/exposure to oth autonomic drug on farm |
| U207y00 | [X]Int self poison oth autonomic drug other spec place |
| U205.00 | [X]Intent self poison/exposure to narcotic drug |
| U204.11 | [X]Overdose - antidepressant |
| U204.12 | [X]Overdose - amitriptyline |
| U204.13 | [X]Overdose - SSRI |
| U208000 | [X]Int self poison/exposure to oth/unsp drug/medicam home |
| U204000 | [X]Int self poison/exposure to psychotropic drug at home |
| U206.00 | [X]Intent self poison/exposure to hallucinogen |
| U205000 | [X]Int self poison/exposure to narcotic drug at home |
| U202z00 | [X]Intent self poison sedative hypnotic unspecif place |
| U204z00 | [X]Intent self poison psychotropic drug unspecif place |
| U205z00 | [X]Intent self poison narcotic drug unspecif place |
| U207.00 | [X]Intent self poison/exposure to oth autonomic drug |
| U207000 | [X]Int self poison/exposure to oth autonomic drug at home |
| U207z00 | [X]Intent self poison oth autonomic drug unspecif place |
| U202y00 | [X]Int self poison sedative hypnotic other spec place |
| U204y00 | [X]Int self poison psychotropic drug other spec place |
| U205y00 | [X]Int self poison narcotic drug other spec place |
| U206000 | [X]Int self poison/exposure to hallucinogen at home |
| U206z00 | [X]Intent self poison hallucinogen unspecif place |
| U208.00 | [X]Int self poison/exposure to other/unspec drug/medicament |
| U208100 | [X]Intent self poison oth/unsp drug/medicam res institut |
| U208200 | [X]Int self poison oth/uns drug/med school/pub admin area |
| U208300 | [X]Int self poison oth/uns drug/med in sport/athletic area |
| U208400 | [X]Intent self pois oth/unsp drug/medic in street/highway |
| U208500 | [X]Intent self pois oth/unsp drug/medic trade/service area |
| U208700 | [X]Int self poison/exposure to oth/unsp drug/medic on farm |
| U209100 | [X]Intent self poison alcohol at res institut |
| U209200 | [X]Int self poison alcohol school/pub admin area |
| U209300 | [X]Int self poison alcohol in sport/athletic area |
| U209400 | [X]Intent self pois alcohol in street/highway |
| U209500 | [X]Intent self pois alcohol trade/service area |
| U209600 | [X]Int self pois alcohol indust/construct area |
| U209700 | [X]Int self poison/exposure to alcohol on farm |
| U20A100 | [X]Int self poison org solvent halogen hydrocarb res instit |
| U20A200 | [X]Int self poison org solvent halogen hydrocarb school |
| U20A300 | [X]Int self poison org solvent halogen hydrocarb sport area |
| U20A500 | [X]Int self poison org solvent halogen hydrocarb trade area |
| U20A600 | [X]Int self pois org solvent halogen hydrocarb indust area |
| U20A700 | [X]Int self poison org solvent halogen hydrocarb on farm |
| U20Ay00 | [X]Int self pois org solv halogen hydrocarp oth spec place |
| U20B100 | [X]Intent self poison other gas/vapour at res institut |
| U20B300 | [X]Int self poison other gas/vapour in sport/athletic area |
| U20B400 | [X]Intent self pois other gas/vapour in street/highway |
| U20B500 | [X]Intent self pois other gas/vapour trade/service area |
| U20B600 | [X]Int self pois other gas/vapour indust/construct area |
| U20B700 | [X]Int self poison/exposure to other gas/vapour on farm |
| U20C000 | [X]Int self poison/exposure to pesticide at home |
| U20C100 | [X]Intent self poison pesticide at res institut |
| U20C200 | [X]Int self poison pesticide school/pub admin area |
| U20C300 | [X]Int self poison pesticide in sport/athletic area |
| U20C400 | [X]Intent self pois pesticide in street/highway |
| U20C500 | [X]Intent self pois pesticide trade/service area |
| U20C600 | [X]Int self pois pesticide indust/construct area |
| U20Cz00 | [X]Intent self poison pesticide unspecif place |
| U20y100 | [X]Intent self poison unspecif chemical at res institut |
| U20y300 | [X]Int self poison unspecif chemical in sport/athletic area |
| U20y400 | [X]Intent self pois unspecif chemical in street/highway |
| U20y500 | [X]Intent self pois unspecif chemical trade/service area |
| U20y600 | [X]Int self pois unspecif chemical indust/construct area |
| U20y700 | [X]Int self poison/exposure to unspecif chemical on farm |
| U20yy00 | [X]Int self poison unspecif chemical other spec place |
| U20B.00 | [X]Intent self poison/exposure to other gas/vapour |
| U20B.11 | [X]Self carbon monoxide poisoning |
| U20y.00 | [X]Intent self poison/exposure to unspecif chemical |
| U20y000 | [X]Int self poison/exposure to unspecif chemical at home |
| U20A.00 | [X]Intentional self poison organ solvent halogen hydrocarb |
| U209z00 | [X]Intent self poison alcohol unspecif place |
| U209000 | [X]Int self poison/exposure to alcohol at home |
| U20C.11 | [X]Self poisoning with weedkiller |
| U20B000 | [X]Int self poison/exposure to other gas/vapour at home |
| U20C.12 | [X]Self poisoning with paraquat |
| U20yz00 | [X]Intent self poison unspecif chemical unspecif place |
| U20C.00 | [X]Intent self poison/exposure to pesticide |
| U20Az00 | [X]Int self pois org solv halogen hydrocarb unspec place |
| U20Bz00 | [X]Intent self poison other gas/vapour unspecif place |
| U208y00 | [X]Int self poison oth/unsp drug/medic other spec place |
| U20A000 | [X]Intent self pois organ solvent halogen hydrocarb home |
| U209y00 | [X]Int self poison alcohol other spec place |
| U20B200 | [X]Int self poison other gas/vapour school/pub admin area |
| U20Cy00 | [X]Int self poison pesticide other spec place |
| U20A400 | [X]Int self poison org solvent halogen hydrocarb in highway |
| U20By00 | [X]Int self poison other gas/vapour other spec place |
| U20C700 | [X]Int self poison/exposure to pesticide on farm |
| U20y200 | [X]Int self poison unspecif chemical school/pub admin area |
| U208600 | [X]Int self pois oth/unsp drug/medic indust/construct area |
| U20A.11 | [X]Self poisoning from glue solvent |
| U214.00 | [X]Intent self harm by hangng strangult/suffoct street/h'way |
| U223.00 | [X]Intent self harm by drown/submersn occ sport/athlet area |
| U224.00 | [X]Intent self harm by drowning/submersn occ street/highway |
| U225.00 | [X]Intent self harm by drown/submersn occ trade/servce area |
| U226.00 | [X]Intent self harm by drown/submers occ indust/constr area |
| U227.00 | [X]Intent self harm by drowning/submersion occurrn on farm |
| U230.00 | [X]Intention self harm by handgun discharge occurrn at home |
| U231.00 | [X]Intent self harm by handgun disch occ in resid instit'n |
| U232.00 | [X]Intent self harm h'gun disch occ sch oth ins/pub adm area |
| U233.00 | [X]Intent self harm by handgun disch occ sport/athlet area |
| U234.00 | [X]Intent self harm by handgun disch occ on street/highway |
| U235.00 | [X]Intent self harm by handgun disch occ trade/service area |
| U236.00 | [X]Intent self harm by handgun disch occ indust/constr area |
| U237.00 | [X]Intention self harm by handgun discharge occurrn on farm |
| U23y.00 | [X]Intent self harm by handgun disch occ at oth specif plce |
| U23z.00 | [X]Intent self harm by handgun disch occ at unspecif place |
| U241.00 | [X]Int self harm rifl s'gun/lrg frarm disch occ resid instit |
| U242.00 | [X]Int slf hrm rifl s'gun/lrg frarm dis sch/ins/pub adm area |
| U243.00 | [X]Int self harm rifl s'gun/lrg frarm disch sprt/athlet area |
| U244.00 | [X]Int self harm rifl s'gun/lrg frarm disch occ street/h'way |
| U245.00 | [X]Int self harm rifl s'gun/lrg frarm disch trad/servce area |
| U246.00 | [X]Int slf hrm rifl s'gun/lrg frarm disch indust/constr area |
| U247.00 | [X]Intent self harm rifle sh'gun/largr firarm disch occ farm |
| U24y.00 | [X]Int self harm rifl s'gun/lrg frarm disch oth specif place |
| U24z.00 | [X]Int self harm rifl s'gun/lrg frarm disch occ unspec place |
| U250.00 | [X]Intent self harm oth/unspecif firearm disch occ at home |
| U251.00 | [X]Intent self harm oth/unsp firearm disch occ resid instit |
| U252.00 | [X]Inten self harm oth/uns firarm disch sch/ins/pub adm area |
| U253.00 | [X]Inten self harm oth/uns firearm disch occ sprt/athl area |
| U254.00 | [X]Intent self harm oth/unsp firearm disch occ street/h'way |
| U255.00 | [X]Intent self harm oth/uns firearm disch trade/servce area |
| U256.00 | [X]Inten self harm oth/uns firearm disch indust/constr area |
| U257.00 | [X]Intent self harm oth/unspecif firearm disch occ on farm |
| U25y.00 | [X]Intent self harm oth/unsp firearm disch oth specif place |
| U25z.00 | [X]Intent self harm oth/unsp firearm disch occ unspecif plce |
| U260.00 | [X]Intention self harm by explosive material occurrn home |
| U261.00 | [X]Intention self harm by explosiv materl occ resid instit |
| U262.00 | [X]Intent self harm by explosiv materl sch/ins/pub adm area |
| U263.00 | [X]Intent self harm by explosv materl occ sport/athlet area |
| U264.00 | [X]Intention self harm by explosiv materl occ street/highway |
| U265.00 | [X]Intent self harm by explosv materl occ trade/servce area |
| U266.00 | [X]Intent self harm by explosv materl occ indust/constr area |
| U267.00 | [X]Intention self harm by explosive material occurrn farm |
| U22..00 | [X]Intentional self harm by drowning and submersion |
| U21z.00 | [X]Intent self harm by hangng strangul/suffoct unspecif plce |
| U24..00 | [X]Intent self harm by rifle shotgun/larger firearm disch |
| U211.00 | [X]Intent self harm by hangng strangult/suffoct resid instit |
| U212.00 | [X]Inten slf harm hang strang/suffc sch oth ins/pub adm area |
| U21y.00 | [X]Intent self harm by hangng strangul/suffoct oth spec plce |
| U22y.00 | [X]Intent self harm by drown/submersn occ oth specif place |
| U22z.00 | [X]Intent self harm by drown/submersn occ unspecified place |
| U220.00 | [X]Intent self harm by drowning/submersion occurrn at home |
| U213.00 | [X]Intent self harm by hang strangl/suffc sport/athlet area |
| U215.00 | [X]Intent self harm by hang strangl/suffc trade/service area |
| U216.00 | [X]Intent self harm by hang strangl/suffc indust/constr area |
| U217.00 | [X]Intent self harm by hanging strangulat/suffocat occ farm |
| U221.00 | [X]Intent self harm by drowning/submersn occ resid instit'n |
| U25..00 | [X]Intent self harm by other/unspecified firearm discharge |
| U222.00 | [X]Intent self harm drown/submers occ sch/ins/pub adm area |
| U23..00 | [X]Intentional self harm by handgun discharge |
| U240.00 | [X]Intent self harm rifle sh'gun/largr firarm disch occ home |
| U26..00 | [X]Intentional self harm by explosive material |
| U210.00 | [X]Intent self harm by hanging strangulat/suffocat occ home |
| U26y.00 | [X]Intent self harm by explosiv materl occ oth specif place |
| U26z.00 | [X]Intent self harm by explosiv materl occ unspecif place |
| U271.00 | [X]Intent self harm by smoke fire/flame occ resid instit'n |
| U272.00 | [X]Intent self harm by smoke fire/flame sch/ins/pub adm area |
| U273.00 | [X]Intent self harm by smok fire/flam occ sport/athlet area |
| U275.00 | [X]Intent self harm by smok fire/flam occ trade/servce area |
| U276.00 | [X]Intent self harm by smok fire/flam occ indust/constr area |
| U277.00 | [X]Intention self harm by smoke fire/flames occurrn on farm |
| U281.00 | [X]Intent self harm by steam hot vapour/obj occ resid instit |
| U283.00 | [X]Int self harm by steam hot vapour/obj occ sport/athl area |
| U284.00 | [X]Intent self harm by steam hot vapour/obj occ street/h'way |
| U285.00 | [X]Int self harm by steam hot vapour/obj trade/service area |
| U286.00 | [X]Int self harm by steam hot vapour/obj indust/constr area |
| U287.00 | [X]Intent self harm by steam hot vapour/hot obj occ on farm |
| U28y.00 | [X]Intent self harm by steam hot vapour/obj oth specif place |
| U293.00 | [X]Intent self harm by sharp object occ sports/athlet area |
| U2A4.00 | [X]Intention self harm by blunt object occ street/highway |
| U2A5.00 | [X]Intent self harm by blunt object occ trade/service area |
| U2A6.00 | [X]Intent self harm by blunt object occ indust/constr area |
| U2A7.00 | [X]Intentional self harm by blunt object occurrence on farm |
| U2Ay.00 | [X]Intention self harm by blunt object occ oth specif place |
| U2B2.00 | [X]Int self harm jump fr high place sch oth ins/pub adm area |
| U2B3.00 | [X]Intent self harm by jump from high place sport/athl area |
| U2B5.00 | [X]Intent self harm by jump from high place trad/servce area |
| U2B7.00 | [X]Intent self harm by jumping from high place occ on farm |
| U2C0.00 | [X]Intent self harm by jump/lying befor moving obj occ home |
| U2C1.00 | [X]Int self harm jump/lying befr mov obje occ resid instit'n |
| U2C2.00 | [X]Int self harm jump/lying bef mov obj sch/ins/pub adm area |
| U2C3.00 | [X]Int self harm jump/lying bef mov obj occ sprt/athlet area |
| U2C5.00 | [X]Int self harm jump/lying bef mov obj occ trad/servce area |
| U2C6.00 | [X]Int self harm jump/lying befr mov obj indust/constr area |
| U290.00 | [X]Intentional self harm by sharp object occurrence at home |
| U29z.00 | [X]Intentional self harm by sharp object occ unspecif place |
| U27..00 | [X]Intentional self harm by smoke fire and flames |
| U2A..00 | [X]Intentional self harm by blunt object |
| U2C..00 | [X]Intent self harm by jumping / lying before moving object |
| U28..00 | [X]Intentional self harm by steam hot vapours / hot objects |
| U29y.00 | [X]Intention self harm by sharp object occ oth specif place |
| U291.00 | [X]Intent self harm by sharp object occ resident instit'n |
| U2B0.00 | [X]Intent self harm by jumping from high place occ at home |
| U270.00 | [X]Intention self harm by smoke fire/flames occurrn at home |
| U2Bz.00 | [X]Int self harm by jump from high place occ unspecif place |
| U280.00 | [X]Intent self harm by steam hot vapour/hot obj occ at home |
| U27z.00 | [X]Intent self harm by smoke fire/flames occ unspecif place |
| U2A3.00 | [X]Intent self harm by blunt object occ sports/athlet area |
| U2B4.00 | [X]Intent self harm by jump from high place occ street/h'way |
| U2A2.00 | [X]Intent self harm blunt obj occ sch oth ins/pub adm area |
| U296.00 | [X]Intent self harm by sharp object occ indust/constr area |
| U2A1.00 | [X]Intent self harm by blunt object occ resident instit'n |
| U27y.00 | [X]Intent self harm by smoke fire/flame occ oth specif plce |
| U2B1.00 | [X]Intent self harm by jump from high place occ resid instit |
| U2By.00 | [X]Int self harm by jump from high place occ oth specif plce |
| U2C4.00 | [X]Int self harm jump/lying befr mov obje occ street/highway |
| U274.00 | [X]Intent self harm by smoke fire/flame occ street/highway |
| U282.00 | [X]Int self harm by steam hot vapor/obj sch/ins/pub adm area |
| U28z.00 | [X]Intent self harm by steam hot vapour/obj occ unspec place |
| U294.00 | [X]Intention self harm by sharp object occ street/highway |
| U295.00 | [X]Intent self harm by sharp object occ trade/service area |
| U297.00 | [X]Intentional self harm by sharp object occurrence on farm |
| U2A0.00 | [X]Intentional self harm by blunt object occurrence at home |
| U2B6.00 | [X]Int self harm by jump from high place indust/constr area |
| U2Az.00 | [X]Intentional self harm by blunt object occ unspecif place |
| U2B..00 | [X]Intentional self harm by jumping from a high place |
| U292.00 | [X]Intent self harm sharp obj occ sch oth ins/pub adm area |
| U2C7.00 | [X]Intent self harm by jump/lying befor moving obj occ farm |
| U2Cz.00 | [X]Int self harm jump/lying bef mov obje occ unspecif place |
| U2D1.00 | [X]Intent self harm by crash motor vehicl occ resid instit'n |
| U2D2.00 | [X]Int self harm crash motor vehicl occ sch/ins/pub adm area |
| U2D3.00 | [X]Intent self harm by crash motor vehicl occ sprt/athl area |
| U2D5.00 | [X]Intent self harm crash motor vehicl occ trade/servce area |
| U2D7.00 | [X]Intent self harm by crash of motor vehicl occurrn on farm |
| U2y2.00 | [X]Intent self harm oth specif mean occ sch/ins/pub adm area |
| U2y3.00 | [X]Intent self harm by oth specif means occ sport/athl area |
| U2y4.00 | [X]Intent self harm by oth specif means occ street/highway |
| U2y5.00 | [X]Intent self harm by oth specif means occ trad/servce area |
| U2y7.00 | [X]Intentionl self harm by oth specif means occurrn on farm |
| U2z3.00 | [X]Intent self harm unspecif means occurrn sport/athlet area |
| U2z4.00 | [X]Intent self harm by unspecif means occurrn street/highway |
| U2z5.00 | [X]Intent self harm unspecif means occurrn trade/servce area |
| U2z6.00 | [X]Intent self harm unspecif mean occurrn indust/constr area |
| U2z7.00 | [X]Intentional self harm by unspecif means occurrn on farm |
| U30..11 | [X]Deliberate drug poisoning |
| U2y0.00 | [X]Intentionl self harm by oth specif means occurrn at home |
| U2zz.00 | [X]Intent self harm by unspecif means occ at unspecif place |
| U2z0.00 | [X]Intentional self harm by unspecif means occurrn at home |
| U2D..00 | [X]Intentional self harm by crashing of motor vehicle |
| U2yz.00 | [X]Intent self harm by oth specif means occ unspecif place |
| U2y1.00 | [X]Intent self harm by oth specif means occ resid instit'n |
| U2yy.00 | [X]Intent self harm oth specif means occ oth specif place |
| U2z2.00 | [X]Intent self harm by unspec mean occ sch/ins/pub adm area |
| U2zy.00 | [X]Intent self harm by unspecif means occ oth specif place |
| U2Dz.00 | [X]Intent self harm by crash motor vehic occ unspecif place |
| U2z1.00 | [X]Intent self harm by unspecif means occurrn resid instit'n |
| U2Cy.00 | [X]Int self harm jump/lying bef mov obje occ oth specif plce |
| U2D0.00 | [X]Intent self harm by crash of motor vehicl occurrn at home |
| U2D4.00 | [X]Intent self harm by crash motor vehicl occ street/highway |
| U2D6.00 | [X]Intent self harm crash motor vehic occ indust/constr area |
| U2Dy.00 | [X]Intent self harm by crash motor vehic occ oth specif plce |
| U2y6.00 | [X]Intent self harm oth specif means occ indust/constr area |
| U2y..00 | [X]Intentional self harm by other specified means |
| U44..00 | [X]Rifle shotgun+larger firearm discharge undetermin intent |
| U45..00 | [X]Other+unspecified firearm discharge undetermined intent |
| U4B..00 | [X]Falling jumping/pushed from high place undeterm intent |
| U4Bz.00 | [X]Fall jump/push frm high plce undt intnt occ unspecif plce |
| U613200 | [X]Overdose of radiation given during therapy |
| U72..00 | [X]Sequel intentn self-harm assault+event of undeterm intent |
| U720.00 | [X]Sequelae of intentional self-harm |
| Z9K3100 | Removing objects that could be used for suicide attempt |
| ZV1B200 | [V]Personal history of self-harm |
| ZX15.00 | Drowning self |
| ZX11500 | Biting own tongue |
| ZX11600 | Biting sides of own cheeks |
| ZX12.00 | Burning self |
| ZX...00 | Self-harm |
| ZX13.11 | Cuts self |
| ZX1..00 | Self-injurious behaviour |
| ZX11200 | Biting own fingers |
| ZX11.11 | Bites self |
| ZX13.00 | Cutting self |
| ZX11.00 | Biting self |
| ZX1..14 | Self-abusive behaviour |
| ZX11100 | Biting own hand |
| ZX13200 | Cutting own throat |
| ZX1..12 | SIB - Self-injurious behaviour |
| ZX11300 | Biting own toes |
| ZX11400 | Biting own arm |
| ZX...11 | Self-damage |
| ZX1L400 | Self-mutilation of eyes |
| ZX1LD00 | [X]Self mutilation |
| ZX1N.00 | Stabbing self |
| ZX1LA00 | Removing own nails |
| ZX1L500 | Enucleation of own eyes |
| ZX14.00 | Damaging own wounds |
| ZX1M.00 | Shooting self |
| ZX1L200 | Self-mutilation of genitalia |
| ZX14300 | Poking fingers into wound |
| ZX1L300 | Self-mutilation of penis |
| ZX1Q.00 | Throwing self in front of train |
| ZX1S.00 | Throwing self onto floor |
| ZX1L600 | Self-mutilation of ears |
| ZX1L811 | Snapping own bones |
| ZX1L800 | Breaking own bones |
| ZX1R.11 | Jumping in front of vehicle |
| ZX1R.00 | Throwing self in front of vehicle |
| SL...15 | Overdose of drug |
| U20..11 | [X]Deliberate drug overdose / other poisoning |
| ZX1..13 | Deliberate self-harm |
| U29..00 | [X]Intentional self harm by sharp object |
| SLHz.00 | Drug and medicament poisoning NOS |
| ZX13100 | Cutting own wrists |
| U21..00 | [X]Intent self harm by hanging strangulation / suffocation |
| U2z..00 | [X]Intentional self harm by unspecified means |
| 1BD5.00 | High suicide risk |
| ZX1G.00 | Scratches self |
| U209.00 | [X]Intent self poison/exposure to alcohol |
| ZX1I.00 | Self-scalding |
| U208z00 | [X]Intent self poison oth/unsp drug/medic unspecif place |
| TK20.00 | Suicide + selfinflicted poisoning by motor veh exhaust gas |
| U204.00 | [X]Intent self poison/exposure to psychotropic drug |
| ZX1L.00 | Self-mutilation |
| U41..00 | [X]Hanging strangulation + suffocation undetermined intent |
| ZX1H100 | Self-strangulation |
| ZX1L100 | Self-mutilation of hands |
| ZX1K.11 | Setting fire to self |
| TKx7.00 | Suicide and selfinflicted injury caustic subst excl poison |
| ZX1J.00 | Self-electrocution |
| ZX1E.00 | Pinching self |
| 8G6Z.00 | Anti-suicide psychotherapy NOS |
| TK54.00 | Suicide and selfinflicted injury by other firearm |
| ZX1K.00 | Self-incineration |
| ZX1H200 | Self-suffocation |
| TKx0100 | Suicide + selfinflicted injury-lying before moving object |
| U203.00 | [X]Intent self poison/exposure to antiparkinson drug |
| ZX1Q.11 | Jumping under train |
| ZX1K.12 | Setting self alight |
| TK05.00 | Suicide + selfinflicted poisoning by drug or medicine NOS |
| ZX16.00 | Gouging own body parts |
| U2E..00 | [X]Self mutilation |
| ZX18.00 | Hanging self |
| ZX1B200 | Jumping from bridge |
| ZX1B.00 | Jumping from height |
| ZX1B100 | Jumping from building |
| ZX1B300 | Jumping from cliff |
| ZX16100 | Gouging own flesh |
| ZX16200 | Gouging own eyes |

Table S1: Baseline Characteristics

|  | **Exposed** | **Unexposed** |
| --- | --- | --- |
| **Number of patients (n)** | 2,491,086 | 3,120,795 |
| **Number of patients with asthma diagnosis (n)** | 472,984 | 289,733 |
| **Number of patients with eczema diagnosis (n)** | 455,348 | 368,631 |
| **Median (IQR) follow-up period (person years)** | 5.10 (2.02 – 9.68) | 4.10 (1.59 – 8.34) |
| **Mean (SD) age at cohort entry (years)** | 39.42 (23.65) | 35.82 (22.18) |
| **Sex (n)** |  |  |
| Male | 1,129,954 | 1,561,600 |
| Female | 1,361,132 | 1,559,195 |
| **Body mass index, n (%)** |  |  |
| Underweight (<18·5 kg/m^2^) | 40,490 (1.63) | 52,183 (1.67) |
| Normal (18·5-24·9 kg/m^2^) | 687,723 (27.61) | 823,813 (26.37) |
| Overweight (25·0-29·9 kg/m^2^) | 541,784 (21.75) | 559,747 (17.94) |
| Obese (>30·0 kg/m^2^) | 348,395 (13.99) | 309,638 (9.92) |
| Not available | 872,694 (35.03) | 1,376,414 (44.10) |
| **Smoking status, n (%)** |  |  |
| Current smoker | 348,532 (13.99) | 474,997 (15.22) |
| Non-current smoker | 1,574,697 (63.21) | 1,681,427 (53.88) |
| Not available | 567,857 (22.80) | 964,371 (30.90) |
| **Drinking status, n (%)** |  |  |
| Current Drinker | 1,266,898 (50.86) | 1,394,009 (44.67) |
| Non-current drinker | 370,169 (14.86) | 383,121 (12.28) |
| Not available | 854,019 (34.28) | 1,343,665 (43.06) |
| **Townsend index, n (%)** |  |  |
| (Least deprived) 1 | 535,228 (21.49) | 657,876 (21.08) |
| 2 | 464,489 (18.65) | 552,836 (17.71) |
| 3 | 453,156 (18.19) | 559,287 (17.92) |
| 4 | 388,862 (15.61) | 487,405 (15.62) |
| 5 | 262,946 (10.56) | 327,192(10.48) |
| Not available | 386,405 (15.51) | 536,199 (17.18) |
| **Ethnicity, n (%)** |  |  |
| White | 1,050,939 (42.19) | 1,102,464 (35.33) |
| Black | 45,742 (1.84) | 51,693 (1.66) |
| South Asian | 66,060 (2.65) | 74,762 (2.40) |
| Mixed | 16,818 (0.68) | 19,759 (0.63) |
| Other | 37,641 (1.51) | 54,566 (1.75) |
| Missing | 1,273,886 (51.14) | 1,817,551 (58.24) |

Table S2: Hazard ratios with 95% confidence intervals (CI) for mental ill health among patients with any atopic or allergic disorder compared to matched unexposed individuals

|  |  | **Number of outcomes** | **Person Years** | **Incidence Rate**  **(per 10,000 person years)** | **Unadjusted Hazard Ratio** | **Adjusted Hazard Ratio** |
| --- | --- | --- | --- | --- | --- | --- |
| **Composite** | **Exposed** | 229,124 | 15,900,000 | 144.13 | 1.23 (95% CI 1.22-1.24, p<0.001) | 1.16 (95% CI 1.15-1.16, p<0.001) |
|  | **Unexposed** | 203,514 | 17,200,000 | 118.20 |  |  |
|  |  |  | | | | |
| **SMI** | **Exposed** | 5,581 | 17,200,000 | 32.46 | 1.00 (95% CI 0.97-1.04, p 0.834) | 0.99 (95% CI 0.96-1.03, p 0.994) |
|  | **Unexposed** | 5,954 | 18,300,000 | 32.46 |  |  |
|  |  |  | | | | |
| **Anxiety** | **Exposed** | 93,848 | 16,700,000 | 56.21 | 1.30 (95% CI 1.29-1.31, p<0.001) | 1.21 (95% CI 1.20-1.23, p<0.001) |
|  | **Unexposed** | 77,388 | 18,000,000 | 43.11 |  |  |
|  |  |  | | | | |
| **Depression** | **Exposed** | 138,658 | 16,400,000 | 84.59 | 1.24 (95% CI 1.23-1.26, p<0.001) | 1.15 (95% CI 1.14-1.16, p<0.001) |
|  | **Unexposed** | 121,378 | 17,700,000 | 68.75 |  |  |
|  |  |  | | | | |
| **Eating disorders** | **Exposed** | 4,188 | 17,200,000 | 2.44 | 1.06 (95% CI 1.01-1.10, p 0.011) | 1.06 (95% CI 1.01-1.10, p 0.013) |
|  | **Unexposed** | 4,295 | 18,300,000 | 2.34 |  |  |
|  |  |  | | | | |
| **OCD** | **Exposed** | 3,565 | 17,200,000 | 2.07 | 1.19 (95% CI 1.13-1.25, p<0.001) | 1.18 (95% CI 1.13-1.24, p<0.001) |
|  | **Unexposed** | 3,191 | 18,400,000 | 1.74 |  |  |
|  |  |  | | | | |
| **Self harm** | **Exposed** | 21,959 | 17,100,000 | 12.83 | 1.00 (95% CI 0.98-1.02, p 0.945) | 1.03 (95% CI 1.01-1.05, p 0.006) |
|  | **Unexposed** | 23,426 | 18,300,000 | 12.83 |  |  |

*Adjusted hazard ratio: adjusted for age, sex, alcohol use, smoking status, body mass index (BMI), Townsend deprivation quintile score, asthma, and eczema at baseline

Table S3: Hazard ratios with 95% confidence intervals (CI) for mental ill health among patients with food allergy only compared to matched unexposed individuals

|  |  | **Number of outcomes** | **Person Years** | **Incidence Rate**  **(per 10,000 person years)** | **Unadjusted Hazard Ratio** | **Adjusted Hazard Ratio** |
| --- | --- | --- | --- | --- | --- | --- |
| **Composite** | **Exposed** | 6,955 | 583, 190 | 119.26 | 1.20 (95% CI 1.16-1.24, p<0.001) | 1.07 (95% CI 1.03-1.11, p<0.001) |
|  | **Unexposed** | 6,409 | 651,196 | 98.42 |  |  |
|  |  |  | | | | |
| **SMI** | **Exposed** | 180 | 617,205 | 29.16 | 1.27 (95% CI 1.03-1.58, p 0.029) | 1.26 (95% CI 1.00-1.59, p 0.046) |
|  | **Unexposed** | 154 | 681,363 | 22.60 |  |  |
|  |  |  | | | | |
| **Anxiety** | **Exposed** | 2,908 | 604,387 | 48.12 | 1.32 (95% CI 1.25-1.39, p<0.001) | 1.14 (95% CI 1.08-1.21, p<0.001) |
|  | **Unexposed** | 2,390 | 671,622 | 35.59 |  |  |
|  |  |  | | | | |
| **Depression** | **Exposed** | 3,692 | 597,935 | 61.75 | 1.16 (95% CI 1.11-1.22, p<0.001) | 1.02 (95% CI 0.97-1.07, p 0.378) |
|  | **Unexposed** | 3,509 | 663,826 | 52.86 |  |  |
|  |  |  | | | | |
| **Eating disorders** | **Exposed** | 263 | 616,739 | 4.26 | 1.17 (95% CI 0.98-1.39, p 0.082) | 1.10 (95% CI 0.91-1.33, p 0.315) |
|  | **Unexposed** | 250 | 680,942 | 3.67 |  |  |
|  |  |  | | | | |
| **OCD** | **Exposed** | 188 | 617,035 | 3.05 | 1.47 (95% CI 1.18-1.83, p 0.001) | 1.28 (95% CI 1.01-1.63, p 0.042) |
|  | **Unexposed** | 138 | 681,327 | 2.03 |  |  |
|  |  |  | | | | |
| **Self harm** | **Exposed** | 917 | 614,055 | 14.93 | 1.03 (95% CI 0.94-1.13, p 0.505) | 0.98 (95% CI 0.89-1.08, p 0.651) |
|  | **Unexposed** | 954 | 677,879 | 14.07 |  |  |

*Adjusted hazard ratio: adjusted for age, sex, alcohol use, smoking status, body mass index (BMI), Townsend deprivation quintile score, asthma, and eczema at baseline

Table S4: Hazard ratios with 95% confidence intervals (CI) for mental ill health among patients with drug allergy only compared to matched unexposed individuals

|  |  | **Number of outcomes** | **Person Years** | **Incidence Rate**  **(per 10,000 person years)** | **Unadjusted Hazard Ratio** | **Adjusted Hazard Ratio** |
| --- | --- | --- | --- | --- | --- | --- |
| **Composite** | **Exposed** | 136,246 | 8,629,804 | 157.88 | 1.34 (95% CI 1.33-1.35, p<0.001) | 1.27 (95% CI 1.26-1.28, p<0.001) |
|  | **Unexposed** | 104,769 | 8,747,397 | 119.77 |  |  |
|  |  |  | | | | |
| **SMI** | **Exposed** | 3,467 | 9,429,027 | 36.77 | 1.07 (95% CI 1.02-1.12, p 0.007) | 1.07 (95% CI 1.02-1.12, p 0.01) |
|  | **Unexposed** | 3,256 | 9,340,622 | 34.86 |  |  |
|  |  |  | | | | |
| **Anxiety** | **Exposed** | 55,508 | **9,120,391** | 60.86 | 1.43 (95% CI 1.41-1.45, p<0.001) | 1.36 (95% CI 1.34-1.37, p<0.001) |
|  | **Unexposed** | 39,018 | 9,136,285 | 42.71 |  |  |
|  |  |  | | | | |
| **Depression** | **Exposed** | 85,637 | 8,920,011 | 96,01 | 1.34 (95% CI 1.33-1.36, p<0.001) | 1.26 (95% CI 1.24-1.27, p<0.001) |
|  | **Unexposed** | 65,211 | 8,964,301 | 72.75 |  |  |
|  |  |  | | | | |
| **Eating disorders** | **Exposed** | 1,834 | 9,434,611 | 1.94 | 1.13 (95% CI 1.06-1.21, p<0.001) | 1.16 (95% CI 1.09-1.24, p<0.001) |
|  | **Unexposed** | 1,648 | 9,347,463 | 1.76 |  |  |
|  |  |  | | | | |
| **OCD** | **Exposed** | 1,663 | 9,434,811 | 1.76 | 1.30 (95% CI 1.21-1.40, p<0.001) | 1.34 (95% CI 1.25-1.45, p<0.001) |
|  | **Unexposed** | 1,268 | 9,348,323 | 1.36 |  |  |
|  |  |  | | | | |
| **Self harm** | **Exposed** | 10,780 | 9,390,771 | 11.48 | 1.13 (95% CI 1.10-1.16, p<0.001) | 1.19 (95% CI 1.15-1.22, p<0.001) |
|  | **Unexposed** | 9,515 | 9,309,052 | 10.22 |  |  |

*Adjusted hazard ratio: adjusted for age, sex, alcohol use, smoking status, body mass index (BMI), Townsend deprivation quintile score, asthma, and eczema at baseline

Table S5: Hazard ratios with 95% confidence intervals (CI) for mental ill health among patients with anaphylaxis only compared to matched unexposed individuals

|  |  | **Number of outcomes** | **Person Years** | **Incidence Rate**  **(per 10,000 person years)** | **Unadjusted Hazard Ratio** | **Adjusted Hazard Ratio** |
| --- | --- | --- | --- | --- | --- | --- |
| **Composite** | **Exposed** | 1,773 | 100,899 | 175.72 | 1.54 (95% CI 1.43-1.65, p<0.001) | 1.42 (95% CI 1.31-1.53, p<0.001) |
|  | **Unexposed** | 1,260 | 108,843 | 115.76 |  |  |
|  |  |  | | | | |
| **SMI** | **Exposed** | 33 | 111,340 | 2.96 | 0.90 (95% CI 0.57-1.44, p 0.675) | 0.89 (95% CI 0.54-1.46. p 0.635) |
|  | **Unexposed** | 38 | 115,815 | 3.28 |  |  |
|  |  |  | | | | |
| **Anxiety** | **Exposed** | 769 | 107,135 | 71.78 | 1.76 (95% CI 1.56-1.97, p<0.001) | 1.61 (95% CI 1.43-1.82, p<0.001) |
|  | **Unexposed** | 462 | 113,477 | 40.71 |  |  |
|  |  |  | | | | |
| **Depression** | **Exposed** | 1,050 | 104,749 | 100.24 | 1.52 (95% CI 1.39-1.67. p<0.001) | 1.40 (95% CI 1.27-1.55, p<0.001) |
|  | **Unexposed** | 747 | 111,561 | 66.96 |  |  |
|  |  |  | | | | |
| **Eating disorders** | **Exposed** | 31 | 111,337 | 2.78 | 1.12 (95% CI 0.67-1.86, p 0.664) | 0.93 (95% CI 0.54-1.63, p 0.813) |
|  | **Unexposed** | 29 | 115,840 | 2.50 |  |  |
|  |  |  | | | | |
| **OCD** | **Exposed** | 32 | 111,288 | 2.87 | 2.18 (95% CI 1.19-3.97, p 0.011) | 2.59 (95% CI 1.38-4.86, p 0.003) |
|  | **Unexposed** | 16 | 115,901 | 1.38 |  |  |
|  |  |  | | | | |
| **Self harm** | **Exposed** | 196 | 110,613 | 17.72 | 1.46 (95% CI 1.18-1.82, p 0.001) | 1.43 (95% CI 1.13-1.80, p 0.003) |
|  | **Unexposed** | 139 | 115,346 | 12.05 |  |  |

*Adjusted hazard ratio: adjusted for age, sex, alcohol use, smoking status, body mass index (BMI), Townsend deprivation quintile score, asthma, and eczema at baseline

Table S6: Hazard ratios with 95% confidence intervals (CI) for mental ill health among patients with urticaria only compared to matched unexposed individuals

|  |  | **Number of outcomes** | **Person Years** | **Incidence Rate**  **(per 10,000 person years)** | **Unadjusted Hazard Ratio** | **Adjusted Hazard Ratio** |
| --- | --- | --- | --- | --- | --- | --- |
| **Composite** | **Exposed** | 43,001 | 2,966,951 | 175.72 | 1.25 (95% CI 1.24-1.27, p<0.001) | 1.16 (95% CI 1.14-1.18, p<0.001) |
|  | **Unexposed** | 35,369 | 3,050,446 | 115.76 |  |  |
|  |  |  | | | | |
| **SMI** | **Exposed** | 863 | 3,231,215 | 2.67 | 0.88 (95% CI 0.80-0.97, p 0.007) | 0.85 (95% CI 0.77-0.93, p 0.001) |
|  | **Unexposed** | 974 | 3,249,189 | 3.00 |  |  |
|  |  |  | | | | |
| **Anxiety** | **Exposed** | 18,675 | 3,123,901 | 59.78 | 1.33 (95% CI 1.30-1.36, p<0.001) | 1.22 (95% CI 1.20-1.25, p<0.001) |
|  | **Unexposed** | 14,051 | 3,176,950 | 44.23 |  |  |
|  |  |  | | | | |
| **Depression** | **Exposed** | 24,988 | 3,072,521 | 81.33 | 1.28 (95% CI 1.25-1.30, p<0.001) | 1.16 (95% CI 1.14-1.18, p<0.001) |
|  | **Unexposed** | 20,079 | 3,132,763 | 64.09 |  |  |
|  |  |  | | | | |
| **Eating disorders** | **Exposed** | 1,090 | 3,229,622 | 3.38 | 1.14 (95% CI 1.05-1.24, p 0.003) | 1.14 (95% CI 1.04-1.24, p 0.004) |
|  | **Unexposed** | 972 | 3,249,245 | 2.99 |  |  |
|  |  |  | | | | |
| **OCD** | **Exposed** | 786 | 3,230,785 | 2.43 | 1.21 (95% CI 1.09-1.35, p<0.001) | 1.18 (95% CI 1.06-1.31, p 0.002) |
|  | **Unexposed** | 645 | 3,250,544 | 1.98 |  |  |
|  |  |  | | | | |
| **Self harm** | **Exposed** | 5,105 | 3,209,184 | 15.91 | 1.05 (95% CI 1.01-1.09, p 0.021) | 1.05 (95% CI 1.01-1.09, p 0.021) |
|  | **Unexposed** | 4,838 | 3,230,221 | 14.98 |  |  |

*Adjusted hazard ratio: adjusted for age, sex, alcohol use, smoking status, body mass index (BMI), Townsend deprivation quintile score, asthma, and eczema at baseline

Table S7: Hazard ratios with 95% confidence intervals (CI) for mental ill health among patients with allergic rhinitis only compared to matched unexposed individuals

|  |  | **Number of outcomes** | **Person Years** | **Incidence Rate**  **(per 10,000 person years)** | **Unadjusted Hazard Ratio** | **Adjusted Hazard Ratio** |
| --- | --- | --- | --- | --- | --- | --- |
| **Composite** | **Exposed** | 104,297 | 7,075,459 | 147.41 | 1.21 (95% CI 1.20-1.22, p<0.001) | 1.12 (95% CI 1.11-1.13, p<0.001) |
|  | **Unexposed** | 95,412 | 7,744,558 | 123.20 |  |  |
|  |  |  | | | | |
| **SMI** | **Exposed** | 2,354 | 7,695,113 | 3.06 | 0.96 (95% CI 0.91-1.02, p 0.169) | 0.96 (95% CI 0.91-1.02, p 0.217) |
|  | **Unexposed** | 2,622 | 8,282,321 | 3.17 |  |  |
|  |  |  | | | | |
| **Anxiety** | **Exposed** | 44,525 | 7,448,442 | 59.78 | 1.30 (95% CI 1.27-1.31, p<0.001) | 1.19 (95% CI 1.18-1.21, p<0.001) |
|  | **Unexposed** | 37,075 | 8,091,138 | 45.82 |  |  |
|  |  |  | | | | |
| **Depression** | **Exposed** | 61,919 | 7,318,942 | 84.60 | 1.22 (95% CI 1.20-1.23, p<0.001) | 1.12 (95% CI 1.11-1.13, p<0.001) |
|  | **Unexposed** | 55,821 | 7,960,109 | 70.13 |  |  |
|  |  |  | | | | |
| **Eating disorders** | **Exposed** | 2,200 | 7,694,901 | 2.86 | 1.06 (95% CI 1.00-1.12, p 0.065) | 1.02 (95% CI 0.96-1.09, p 0.506) |
|  | **Unexposed** | 2,284 | 8,284,274 | 2.76 |  |  |
|  |  |  | | | | |
| **OCD** | **Exposed** | 1,972 | 7,695,515 | 2.56 | 1.23 (95% CI 1.15-1.31, p<0.001) | 1.18 (95% CI 1.10-1.26, p<0.001) |
|  | **Unexposed** | 1,726 | 8,286,295 | 2.08 |  |  |
|  |  |  | | | | |
| **Self harm** | **Exposed** | 10,787 | 7,652,323 | 14.10 | 0.93 (95% CI 0.91-0.95, p<0.001) | 0.93 (95% CI 0.91-0.96, p<0.001) |
|  | **Unexposed** | 12,491 | 8,234,424 | 15.17 |  |  |

*Adjusted hazard ratio: adjusted for age, sex, alcohol use, smoking status, body mass index (BMI), Townsend deprivation quintile score, asthma, and eczema at baseline
