# Supplementary material for: Association between atopic disorders and mental ill health: a UK-based retrospective cohort study
Source: BMJ Open. 2025 May 31;15(5):e089181. doi: 10.1136/bmjopen-2024-089181 (PMC12142133; doi:10.1136/bmjopen-2024-089181)
Supplement: Supplementary file 3 [file bmjopen-2024-089181.reviewer_comments.pdf]

PEER REVIEW HISTORY

BMJ Open publishes all reviews undertaken for accepted manuscripts. Reviewers are asked to complete a checklist review form and are provided with free text boxes to elaborate on their assessment. These free text comments are reproduced below.

ARTICLE DETAILS

Title (Provisional)

The association between atopic disorders and mental ill health: a UK-based retrospective cohort study

Authors

Minhas, Sonica; Chandan, Joht Singh; Knibb, Rebecca; Diwakar, Lavanya; Adderley, Nicola

---

VERSION 1 - REVIEW

**Reviewer**	**1**
**Name**	**Polloni, Laura**
**Affiliation**	**Padua University Hospital**
**Date**	**06-Jul-2024**
**COI**	**None**

This study aims to examine the mental ill health burden associated with allergic and atopic disorders, in a UK primary care cohort. The topic is timely, interesting and offers original issues. As there is already a strong evidence base for asthma, allergic rhinitis and atopic dermatitis the authors focused the analysis on food allergy, drug allergy, anaphylaxis, urticaria, allergic rhinoconjunctivitis.

The strengths of this open, retrospective, population-based UK cohort study are a large sample, control sample and medical diagnosis. However, there are some clarifications and adjustments to be made. Please consider the following comments to improve the manuscript.

1. Abstract: in participants section it would be useful reporting participants age (mean or range).

2. As atopic and allergic disorders the authors rightly report food allergy, drug allergy, anaphylaxis, urticaria, and allergic rhino-conjunctivitis. Anaphylaxis is usually caused by food allergy, drug allergy or hymenoptera venom allergy (in addition to idiopathic cases and exercise anaphylaxis). Why was it decided to consider it as a separate category?

3. P. 6 lines 16-18: What should be “common mental disorders”? Do they also include severe mental illness or are they two opposing categories? It is not clear. Anxiety and depression can also resulting in functional and occupational impairment, limiting major life activities.

4. Patient and Public Involvement section is not very clear.

5. Self-harm should be defined in more detail. Do you mean nonsuicidal self-injury disorder (NSSID)?

6. Although the data is anonymized, it is not clear to me whether the participants gave their consent for it to be used in the research and were informed about the objective of the study.

7. Discussion: Anaphylaxis exposure had the greatest risk of subsequent mental health diagnosis, with 43% increased risk compared to those without. They were most likely to be diagnosed with OCD, anxiety, or depression (137%, 59% and 36% increased risk respectively). Patients who have suffered anaphylaxis may also experience post-traumatic stress disorder. Why was this disorder not taken into consideration?

**Reviewer**	**2**
**Name**	**Protudjer, Jennifer**
**Affiliation**	**University of Manitoba, Pediatrics and Child Health**
**Date**	**26-Mar-2025**
**COI**	**I declare that I have no competing interests.**

Thank you for the opportunity to review the manuscript titled “The association between atopic disorders and mental ill health: a UK-based retrospective cohort study,” which was submitted to BMJ Open as original research. Therein, the authors aimed to “examine the mental ill health burden associated with allergic and atopic disorders, in a UK primary care cohort” (copied verbatim from the abstract). This is a registry study that made use of nearly 2.5 million individuals with primary-care recorded atopic diseases, and age, sex and deprivation score to a approximately 3.1 individuals. This is an important and interesting analyses rooted in rigorously collected data, and which may serve to inform interprofessional healthcare delivery for those living with allergic conditions. I have only minor comments.

1. Abstract: Please clarify what is a Townsend deprivation score. The same comment applies to the Methods in the main text.

2. Abstract: For clarity, please confirm that mental ill health was (presumably) identified via ICD-10 codes. The same comment applies to the Methods in the main text.

3. Abstract: What were the regression models adjusted for? This should be detailed in the Abstract, Methods.

4. Introduction, Line 9: Please introduce the abbreviation, IgE. This particularly important as the journal is not allergy-specific/focused.

5. The authors performed many analyses. This raises two comments. First, was a Bonferroni correction (or similar) applied? Second, it is a pity that more tables could not be included in the main manuscript.

VERSION 1 - AUTHOR RESPONSE

Comments from Reviewer #1

Comment 1

Abstract: in participants section it would be useful reporting participants age (mean or range).

Response 1

We have reported the mean age with standard deviation of exposed and unexposed patients in the participants section of the abstract.

Comment 2

As atopic and allergic disorders the authors rightly report food allergy, drug allergy, anaphylaxis, urticaria, and allergic rhino-conjunctivitis. Anaphylaxis is usually caused by food allergy, drug allergy or hymenoptera venom allergy (in addition to idiopathic cases and exercise anaphylaxis). Why was it decided to consider it as a separate category?

Response 2

We agree that with respect to aetiology anaphylaxis is a result of the above-mentioned disorders. However, for the purposes of this analysis, we chose to consider anaphylaxis as a distinct category because of its unique clinical presentation and severity, and therefore potential for a distinct profile of psychological impact and mental health consequences. Also, while anaphylaxis has bespoke codes within the THIN database, the underlying cause is not specifically recorded.

Comment 3

P. 6 lines 16-18: What should be “common mental disorders”? Do they also include severe mental illness or are they two opposing categories? It is not clear. Anxiety and depression can also resulting in functional and occupational impairment, limiting major life activities.

Response 3

Common mental disorders (CMDs) refer to conditions which interfere with daily functioning, but do not typically impair insight or cognition. Therefore, this typically

includes disorders such as depression, anxiety and panic disorder. Whilst we agree, that depression and anxiety can also result in major limitations of life activities, severe mental illness (SMI) in this context is used to refer to psychoses and bipolar disorder, as per the referenced studies and clinical coding in the database used for this study (Methods section; line 227-228). We have edited the section as below:

CMDs comprise of mental health disorders that interfere with daily functioning, but not insight or cognition. This includes different types of depression and anxiety, mixed depression-anxiety, phobias, obsessive compulsive disorder (OCD) and panic disorder.[1] Mental health disorders are an important cause of disability and are a risk factor for premature mortality.[2,3] Severe mental illness (SMI), by contrast, is a subset of mental health disorders characterized by a greater degree of functional and occupational impairment, limiting major life activities and typically includes psychoses and bipolar disorder.[4]

Comment 4

Patient and Public Involvement section is not very clear.

Response 4

Due to use of de-identified data, there was no patient and/or public involvement in this study.

This section has been edited to read as:

Patients and/or the public were not involved in setting the research or interpretation and write up of results. This was deemed unnecessary as all patient related data used was anonymised.

Comment 5

Self-harm should be defined in more detail. Do you mean nonsuicidal self-injury disorder (NSSID)?

Response 5

In this analysis, self-harm has a broader definition (and as demonstrated by the Read codes used, see Appendix B, page 20) includes any deliberate behaviours inflicting self-harm, regardless of the intent and/or presence of suicidal thoughts. Therefore, this includes a spectrum of outcomes from suicide to nonsuidical self-injury. We had refined the Methods to clarify this:

The aim of this study was to compare the risk of mental ill health (composite measure: defined through Read codes describing depression, anxiety, severe mental illness [psychosis, bipolar disorder or schizophrenia], eating disorders, OCD, and self-harm) in exposed patients (Read codes for food allergy, drug allergy, anaphylaxis, urticaria, allergic rhino-conjunctivitis) with unexposed patients (those

without such codes). For this analysis, self-harm includes any deliberate act of inflicting harm on oneself, regardless of suicidal intent.

Comment 6

Although the data is anonymized, it is not clear to me whether the participants gave their consent for it to be used in the research and were informed about the objective of the study.

Response 6

The data for this study are derived from the IQVIA Medical Research Database (IMRD) which contains de-identified electronic medical records from participating UK primary care general practices. All individuals registered with a GP in the UK are given the option of opting out of data collection and hence consent is implied. These data are provided by patients as a part of their routine primary care and since it is de-identified, there is no opportunity for the research team to seek independent written consent from the patients contributing to the electronic research database.

Comment 7

Discussion: Anaphylaxis exposure had the greatest risk of subsequent mental health diagnosis, with 43% increased risk compared to those without. They were most likely to be diagnosed with OCD, anxiety, or depression (137%, 59% and 36% increased risk respectively). Patients who have suffered anaphylaxis may also experience post-traumatic stress disorder. Why was this disorder not taken into consideration?

Response 7

PTSD was not included as an outcome due to limitations in its clinical recording in primary care. Firstly, there is significant overlap between depression and PTSD which means that in general practice settings it can both be underdiagnosed and non-specifically coded. Secondly, PTSD can be underrecognized and therefore underdiagnosed in primary care settings in the UK, especially if there is no referral or follow-up with mental health care. However, we recognize this as an important outcome to explore to delineate the psychological burden associated with atopic disorders.

We have included this as a limitation and recommendation for future research:

Additionally, whilst we recognized post-traumatic stress disorder as an important outcome, we could not include it in this analysis due to underdiagnosis in UK primary care settings and subsequent inconsistent coding in the database. [5]

Further longitudinal studies of large community-representative samples are required to further explore the association between the broad range of atopic disorders and mental health disorders, including post-traumatic stress disorder.

Comments from Reviewer #2

Comment 1

Abstract: Please clarify what is a Townsend deprivation score. The same comment applies to the Methods in the main text.

Response 1

The abstract has been revised to indicate that the Townsend deprivation score is a measure of socioeconomic deprivation:

"…matched by sex, age (± 2 years), and socio-economic deprivation (Townsend quintile score) at index".

The Methods in the main text include a detailed description of the Townsend index (lines 225-228):

"The Townsend deprivation quintile score is a measure of socioeconomic status derived from national census data, including variables such as home ownership and employment.[6] A higher score correlates to greater socio-economic deprivation."

Comment 2

Abstract: For clarity, please confirm that mental ill health was (presumably) identified via ICD-10 codes. The same comment applies to the Methods in the main text.

Response 2

Exposures and outcomes of interest have been identified in this study using Read codes. Our data source is the IQVIA Medical Research Database (IMRD) which contains de-identified electronic medical records from participating UK primary care general practices. Patient data, including clinical data, is recorded in this using Read codes which are a hierarchical clinical coding system developed in the 1980s. These are not based directly on ICD-10 codes although there is overlap in the clinical conditions being classified.

The abstract has been amended to clarify this:

The primary outcome was a composite of mental ill health (severe mental illness, anxiety, depression, eating disorders, obsessive-compulsive disorder [OCD], and self-harm), identified using Read codes.

In the methods, we explain our use of Read codes (see lines 201-203; 230-238). Appendix B provides the Read codes used for this study for each exposure and outcome. Although, for clarity, we have included this edit for clarity:

Patient data regarding symptoms, examination findings, and diagnoses is recorded using Read codes [7]- a hierarchical clinical coding system used in UK primary care.

Comment 3

Abstract: What were the regression models adjusted for? This should be detailed in the Abstract, Methods.

Response 3

We have edited the main outcome measures section in the abstract to list the covariates which were adjusted for in the regression analysis:

"Covariates adjusted for were age, sex, alcohol use, smoking status, body mass index (BMI), Townsend deprivation quintile score, asthma exposure, and eczema exposure at baseline."

In the Methods, we have a section under the heading "Study covariates" which details the covariates included in our regression analysis, along with the rationale for their selection (lines 242-254)

Comment 4

Introduction, Line 9: Please introduce the abbreviation, IgE. This particularly important as the journal is not allergy-specific/focused.

Response 4

Thank you for bringing our attention to this. This has been modified to introduce the abbreviation.

Comment 5

The authors performed many analyses. This raises two comments. First, was a Bonferroni correction (or similar) applied? Second, it is a pity that more tables could not be included in the main manuscript.

Response 5

We have added in p-values to all our tables. For all statistically significant results, our p-values are very small therefore applying a Bonferroni correction is unlikely to result in significant difference. Furthermore, all the exposures and outcomes included in our study are inter-related. While applying the Bonferroni correction would reduce the risk of type I error, this would be at the expense of potentially introducing a greater risk of type II error, potentially obscuring meaningful associations.

With respect to the second point, we are not able to include more tables in the main manuscript due to the formatting policies of the journal.

References

1 McManus S, Bebbington P, Jenkins R, Brugha T. Mental Health and Wellbeing in England: Adult Psychiatric Morbidity Survey 2014. Leeds, 2016.

2 Rehm J, Shield KD. Global Burden of Disease and the Impact of Mental and Addictive Disorders. *Curr Psychiatry Rep* 2019; **21**: 10.

3 Charlson FJ, Baxter AJ, Dua T, Degenhardt L, Whiteford HA, Vos T. Excess mortality from mental, neurological and substance use disorders in the Global Burden of Disease Study 2010. *Epidemiol Psychiatr Sci* 2015; **24**: 121–40.

4 Public Health England. Severe mental illness (SMI) and physical health inequalities: briefing. London, 2018.

5 Ehlers A, Gene-Cos N, Perrin S. Low Recognition of Posttraumatic Stress Disorder in Primary Care. *London J Prim Care* 2009; **2**: 36–42.

6 Office for National Statistics, National Records of Scotland, Northern Ireland Statistics and Research Agency, UK Data Service. 2011 UK Townsend Deprivation Scores . 2011. https://statistics.ukdataservice.ac.uk/dataset/2011-uk-townsend-deprivation-scores (accessed Sept 12, 2022).

7 Booth N. What are the Read Codes? *Health Libr Rev* 1994; **11**: 177–82.

VERSION 2 - REVIEW

**Reviewer**	**2**
**Name**	**Protudjer, Jennifer**
**Affiliation**	**University of Manitoba, Pediatrics and Child Health**
**Date**	**06-May-2025**
**COI**	

Thanks to the authors for carefully addressing the original reviewer comments. I have nothing further at this time.
